# Hierarchical Heuristic Species Delimitation Under the Multispecies Coalescent Model with Migration

**DOI:** 10.1093/sysbio/syae050

**Published:** 2024-08-24

**Authors:** Daniel Kornai, Xiyun Jiao, Jiayi Ji, Tomáš Flouri, Ziheng Yang

**Affiliations:** Department of Genetics, Evolution, and Environment, University College London, Gower Street, London WC1E 6BT, UK; Department of Statistics and Data Science, China Southern University of Science and Technology, Shenzhen, Guangdong 518055, China; Department of Genetics, Evolution, and Environment, University College London, Gower Street, London WC1E 6BT, UK; Department of Genetics, Evolution, and Environment, University College London, Gower Street, London WC1E 6BT, UK; Department of Genetics, Evolution, and Environment, University College London, Gower Street, London WC1E 6BT, UK

**Keywords:** BPP, genealogical divergence index, gene flow, giraffes, milksnakes, multispecies coalescent, species delimitation, sunfish

## Abstract

The multispecies coalescent (MSC) model accommodates genealogical fluctuations across the genome and provides a natural framework for comparative analysis of genomic sequence data from closely related species to infer the history of species divergence and gene flow. Given a set of populations, hypotheses of species delimitation (and species phylogeny) may be formulated as instances of MSC models (e.g., MSC for 1 species versus MSC for 2 species) and compared using Bayesian model selection. This approach, implemented in the program bpp, has been found to be prone to over-splitting. Alternatively, heuristic criteria based on population parameters (such as population split times, population sizes, and migration rates) estimated from genomic data may be used to delimit species. Here, we develop hierarchical merge and split algorithms for heuristic species delimitation based on the genealogical divergence index (gdi) and implement them in a Python pipeline called hhsd. We characterize the behavior of the gdi under a few simple scenarios of gene flow. We apply the new approaches to a dataset simulated under a model of isolation by distance as well as 3 empirical datasets. Our tests suggest that the new approaches produced sensible results and were less prone to oversplitting. We discuss possible strategies for accommodating paraphyletic species in the hierarchical algorithm, as well as the challenges of species delimitation based on heuristic criteria.

Delineation of species boundaries is important for characterizing patterns of biological diversity and guiding conservation policy and practice, particularly during the current global changes in climate and environment. Traditionally, species were identified and distinguished using morphological characteristics. The value of genetic data to species delimitation and identification has long been recognized (e.g., Bateson 1909) as genetic data are informative about many related processes, such as species/population divergence and interspecific hybridization (Fujita et al. 2012). Early methods that use genetic data to identify and delimit species relied on simple genetic-distance cutoffs (such as the “4×” or “10×” rules), requiring interspecific divergence to be a few times greater than intraspecific diversity (Hebert et al. 2003, 2004), or reciprocal monophyly in gene trees (Baum and Shaw 1995) (see, e.g., Sites and Marshall 2003 for a review). However, such criteria may be too simplistic as they do not accommodate polymorphism in ancestral populations and incomplete lineage sorting (Hudson and Turelli 2003) or uncertainties in gene-tree reconstruction (Knowles and Carstens 2007; Yang and Rannala 2017).

While genetic and genomic data are clearly informative concerning the species status of populations, interpretation of this evidence may require a proper statistical inference framework. The processes of biological reproduction and accumulation of mutations in the sequences are highly stochastic, as are the sampling errors due to finite amounts of data. The multispecies coalescent (MSC) model (Rannala and Yang 2003) provides a framework for analysis of genomic sequence data from closely related species or populations to infer the order and timings of species/population divergences. Likelihood-based implementations of the MSC accommodate incomplete lineage sorting and stochastic variation in gene trees (so that reciprocal monophyly is not needed) as well as phylogenetic uncertainties at each locus (so that one does not have to rely on inferred gene trees), making it possible to infer population histories even when there is widespread incomplete lineage sorting and there is very little phylogenetic information at every locus (Xu and Yang 2016; Jiao et al. 2021). The MSC model has also been extended to accommodate gene flow between species or populations, assuming either a major hybridization/introgression event at a particular time point in the MSC-with-introgression (MSC-I) model (Wen and Nakhleh 2018; Zhang et al. 2018; Flouri et al. 2020) or continuous migration over an extended time period in the MSC-with-migration (MSC-M) model (Nielsen and Wakeley 2001; Gronau et al. 2011; Hey et al. 2018; Flouri et al. 2023). As hybridization appears to occur commonly in both plants and animals (e.g., *Arabidopsis*, Arnold et al. 2016; *Anopheles* mosquitoes, Fontaine et al. 2015; *Panthera* cats, Figueiro et al. 2017; and Hominins, Nielsen et al. 2017), it may be important to consider explicitly gene flow in speciesdelimitation.

## Species Delimitation Through Comparison of MSC Models

Given a set of populations, different species delimitations correspond to different ways of grouping populations into species. Each species delimitation, together with the phylogeny, for the delimited species can be formulated as an instance of the MSC model and fitted to genomic sequence data sampled from the extant species or populations. Competing models of delimitation can thus be compared via Bayesian model selection using posterior model probabilities or Bayes factors (Yang and Rannala 2010; Ji et al. 2023). In the Bayesian program bpp, this is accomplished by using a Markov chain Monte Carlo (MCMC) algorithm to calculate the posterior probabilities for different MSC models (Yang and Rannala 2010, 2014; Yang 2015; Flouri et al. 2018). In simulations (Luo et al. 2018), bpp showed lower rates of species overestimation and underestimation than the generalized mixed Yule-coalescent method (Pons et al. 2006; Fujisawa and Barraclough 2013) or the Poisson tree process method (Zhang et al. 2013). The approach of model selection appears to be particularly effective in identifying sympatric cryptic species. For example, Ramirez-Reyes et al. (2020) identified 13 new species of leaf-toed geckos in a lineage that diverged30 Ma.

The approach of model selection as implemented in bpp has often been noted to identify more lineages as distinct species than many other methods, especially when applied to geographical populations or races (Sukumaran and Knowles 2017). For example, Campillo et al. (2020) analyzed 99 population pairs in the genus *Drosophila* and found that bpp identified 80 pairs as distinct species, whereas reproductive isolation was identified in only 69 pairs. Similarly, Bamberger et al. (2022) studied 48 *Albinaria cretensis* land snail populations, and found that morphological classifications suggested 3–9 species while bpp suggested 45–48. Barley et al. (2018) simulated multiple populations from a single species that exhibits population structure and isolation by distance, and found that bpp delimited geographically separated populations as distinct species. These studies suggest that the lineages identified by bpp sometimes correspond to populations rather than species (Chambers and Hillis 2020), raising concerns about the apparent over-splitting of bpp (MacGuigan et al. 2021).

## Empirical Species Delimitation Based on Population Parameters

Rather than treating species delimitation as a model-selection problem, an alternative approach is to define species status using an empirical criterion based on parameters that characterize the history of population divergence and gene flow, such as the population split time ($T_{AB}$, in generations), effective population sizes ($N_{A},N_{B}$), and migration rates ($M_{AB}$ and $M_{BA}$, in expected number of migrants per generation). This appears to be a natural approach to take if one recognizes the arbitrariness in species status of allopatric populations. Population parameters can be estimated under the MSC from genomic data, with the stochastic fluctuation of the coalescent process and the phylogenetic uncertainty in genealogical trees accommodated (Jiao et al. 2021).

**Figure 1:**
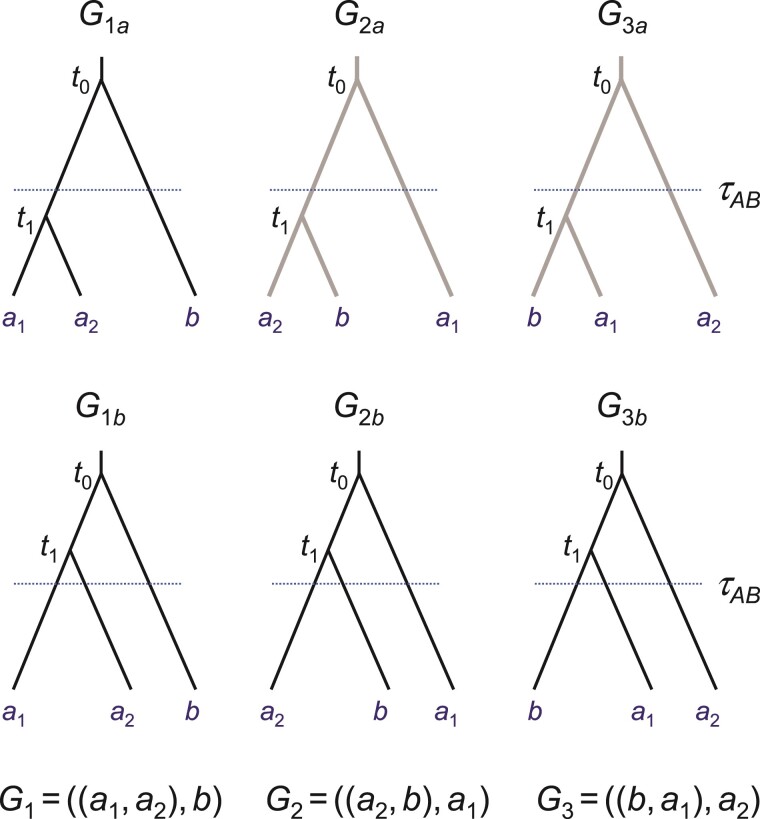
Three possible gene trees for a locus with 2 A sequences and 1 B sequence: $G_{1}=\left(\left(a_{1},a_{2}\right),b\right)$; $G_{2}=\left(\left(a_{2},b\right),a_{1}\right)$; and $G_{3}=\left(\left(b,a_{1}\right),a_{2}\right)$. If the first coalescence (occurring at time $t_{1}$) is more recent than the population divergence ($\tau_{AB}$), the gene trees are labelled $G_{1a},G_{2a}$, and $G_{3a}$; otherwise they are labelled $G_{1b},G_{2b}$, and $G_{3b}$. Note that if there is no gene flow between A and B gene trees $G_{2a}$ and $G_{3a}$ (grayed out) are impossible.

Jackson et al. (2017) introduced such a criterion, called the *genealogical divergence index* (gdi), by considering the probability that 2 sequences sampled from population A ($a_{1}$ and $a_{2}$) coalesce before either of them coalesces with a sequence (b) sampled from population B (Fig. 1). When $a_{1}$ and $a_{2}$ coalesce first, the resulting gene tree has the topology $G_{1}=\left(\left(a_{1},a_{2}\right),b\right)$. Let its probability be $P_{1}=P\left(G_{1}\right)$. In the case of no gene flow between A and B, this is given as


$$P_{1}=1-\frac{2}{3}\,e^{-2\tau_{AB}/\theta_{A}}=1-\frac{2}{3}\,e^{-T_{AB}/2N_{A}}.$$ (1)

The parameter vector is $\Theta =\left(\tau_{AB},\theta_{A},\theta_{B},\theta_{AB}\right)$, with $\tau_{AB}=T_{AB}\mu$ and $\theta_{A}=4N_{A}\mu$, where $T_{AB}$ is the population split time in generations, $N_{A}$ is the population size of A, and μ is the mutation rate per site per generation. Both $\tau_{AB}$ and $\theta_{A}$ are measured in expected number of mutations per site. $P_{1}$ is a simple function of $2\tau_{AB}/\theta_{A}=T_{AB}/\left(2N_{A}\right)$, which is known as branch length in coalescent units since it takes on average $2N_{A}$ generations for 2 sequences from population A to coalesce. As $P_{1}$ ranges from $\frac{1}{3}$ (at $\tau_{AB}=0$, when populations A and B are at panmixia) to 1 (at $\tau_{AB}\to \infty$, when A and B are completely isolated), Jackson et al. (2017) rescaled it so that the resulting index rangesfrom 0 to 1:


$$gdi=\frac{P_{1}-\frac{1}{3}}{1-\frac{1}{3}}=1-e^{-2\tau_{AB}/\theta_{A}}=1-e^{-T_{AB}/2N_{A}}.$$ (2)

Based on a meta-analysis of data from Pinho and Hey (2010), Jackson et al. (2017) suggested the rule of thumb that populations A and B should be considered a single species if gdi<0.2, or 2 distinct species if gdi>0.7. Intermediate values (0.2<gdi<0.7) indicate ambiguous species status. Note that from Equation (2), gdi=0.2 and 0.7 correspond to gene-tree probabilities $P\left(G_{1}\right)=0.47$ and 0.8, respectively, or to split times $T_{AB}/\left(2N_{A}\right)=0.22$ and 1.20 coalescent units, respectively.

Leaché et al. (2019) described a hierarchical merge algorithm for species delimitation based on gdi. Given a set of populations and a guide tree for them, the procedure attempts to merge, progressively, 2 populations into 1 species, judged by gdi. Here, we develop a python pipeline to automate the procedure, called Hierarchical Heuristic Species Delimitation (hhsd). We include a hierarchical split algorithm as well. The hierarchical procedure of Leaché et al. (2019) relied on the MSC model without gene flow. In our pipeline, we account for gene flow by using the MSC-M model implemented recently in bpp (Flouri et al. 2023).

We first discuss the definition and computation of gdi under the MSC-M model, and then describe the algorithms implemented in hhsd. We examine the behavior of the gdi under several simple models of gene flow. We demonstrate our pipeline by analyzing a dataset simulated under an isolation-by-distance model, both under the MSC model with no gene flow and under the MSC-M model accommodating gene flow. Finally, we apply the pipeline to 3 empirical datasets, for giraffes, milksnakes, and sunfish and discuss the results in relation to existing delimitations.

## Theory and Methods

### Redefining the gdi to accommodate complex migration patterns

**Figure 2:**
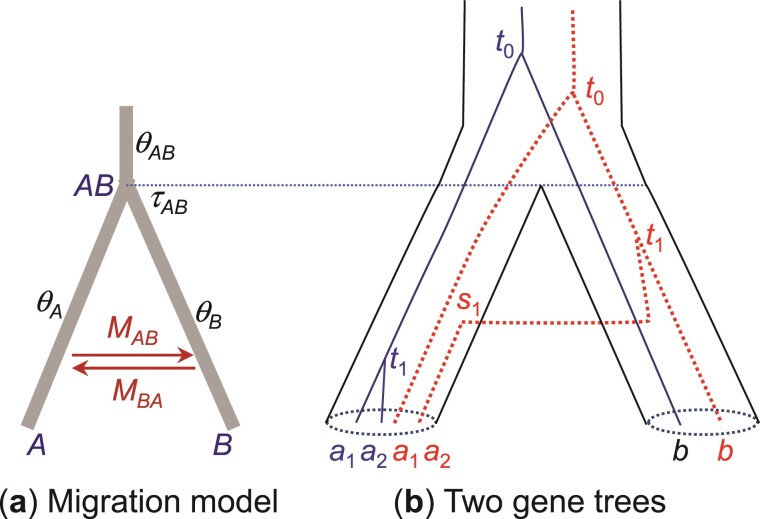
a) An MSC-M model for 2 species or populations (A,B) showing the parameters. The 2 populations diverged time $\tau_{AB}\equiv \tau$ ago and have since been exchanging migrants at the rate of $M_{AB}=m_{AB}N_{B}$ migrants per generation from A to B (under the real-world view with time running forward) and at the rate $M_{BA}=m_{BA}N_{A}$ from B to A. b) Two gene trees, each for 2 A sequences and 1 B sequence ($a_{1},a_{2},b$). In the blue tree (solid lines), $a_{1}$ and $a_{2}$ coalesce first (at time $t_{1}$), in population A, resulting in the gene tree $G_{1}=\left(\left(a_{1},a_{2}\right),b\right)$. This is $G_{1a}$ of Figure 1. In the red tree (dotted lines), $a_{2}$ “migrates” (i.e., is traced back) into B at time $s_{1}$ and coalesces with b in B at time $t_{1}$, resulting in the gene tree $G_{2}=\left(\left(a_{2},b\right),a_{1}\right)$. This is $G_{2a}$ of Figure 1.

The definition of Equation (2) works when populations A and B are completely isolated with no gene flow. When A and B exchange migrants, the gene trees can be modelled using the migration (MSC-M) model, with 6 parameters, Θ=$(\tau_{AB},\theta_{A},\theta_{B},\theta_{AB},M_{AB}$, and $M_{BA})$ (Fig. 2a). Similarly to the case of no gene flow, Jackson et al. (2017) defined $P_{1}=P(G_{1}\left|\Theta \right)$ to be the probability of gene tree $G_{1}$, and rescaled it to define the gdi as


$$gdi_{\text{J}}=\frac{P_{1}-\min\left(P_{1}\right)}{\max\left(P_{1}\right)-\min\left(P_{1}\right)}.$$ (3)

The limits $\min\left(P_{1}\right)=1/3$ and $\max\left(P_{1}\right)=1$ are used in the CalculateGdi function in phrapl (Jackson et al. 2017), which estimates $P_{1}$ by using Hudson’s (2002)ms program to simulate gene trees. When there is gene flow the minimum value achievable by $P_{1}$ depends on the migration events allowed in the model and on how the parameters in the model change, and it is possible for $P_{1}$ to be <1/3, in which case the definition of Equation (3) with $\min\left(P_{1}\right)=1/3$ leads to negative gdi values. We describe 2 such scenarios below.

One approach to dealing with negative gdi values is to set them to 0. Another is to modify the definition of Jackson et al. (2017). We note that with no gene flow, Equation (2) is simply the probability for gene tree $G_{1a}$ (Fig. 1), or the probability that the first coalescence is between $a_{1}$ and $a_{2}$ and that this coalescence occurs before population split when we trace the genealogy of the 3 sequences backwards in time. In other words, we may define gdi as


$$gdi_{\text{K}}=P(G_{1a}\left|\Theta \right)$$ (4)

under both the MSC model with no gene flow and the MSC-M model with gene flow. There is then no need for rescaling as $\mathbb{P}\left(G_{1a}\right)$ ranges from 0 to 1. This definition is expected to work if A and B are non-sister lineages, and if there is gene flow from other populations into either A or B (see below for examples). The definition may also work if gene flow occurs in pulses as in the MSC-I model (Flouri et al. 2020), although this is not pursued here. With no gene flow, the 2 definitions ($gdi_{\text{J}}$ and $gdi_{\text{K}}$) are equivalent but they may differ if there is gene flow.

An ambiguity arises when gdi can be calculated with reference both to A (using aab data or sequences $a_{1},a_{2},b$) and to B (using abb data or sequences $a,b_{1},b_{2}$), leading to 2 indexes,


$$\begin{array}{c} gdi_{A}=1-e^{-2\tau_{AB}/\theta_{A}}=1-e^{-T_{AB}/2N_{A}},\\ gdi_{B}=1-e^{-2\tau_{AB}/\theta_{B}}=1-e^{-T_{AB}/2N_{B}} \end{array}$$ (4)

in the case of no gene flow (cf Equation (2)). If $N_{A}\ll N_{B}$, population A may appear to be a distinct species from B judged by $gdi_{A}$, but B may not appear to be a distinct species from A according to $gdi_{B}$ (Leaché et al. 2019). Another major factor for such conflicting gdi indexes is the asymmetry in gene flow ($M_{AB}\neq M_{BA}$; see below). In our implementation, a merge is accepted if either $gdi_{A}$ or $gdi_{B}$ is less than the cut-off (0.2), whereas in the split algorithm, the split is accepted if both indexes are >0.5 and at least one of them is >0.7.

### The Hierarchical Merge and Split Algorithms

**Figure 3:**
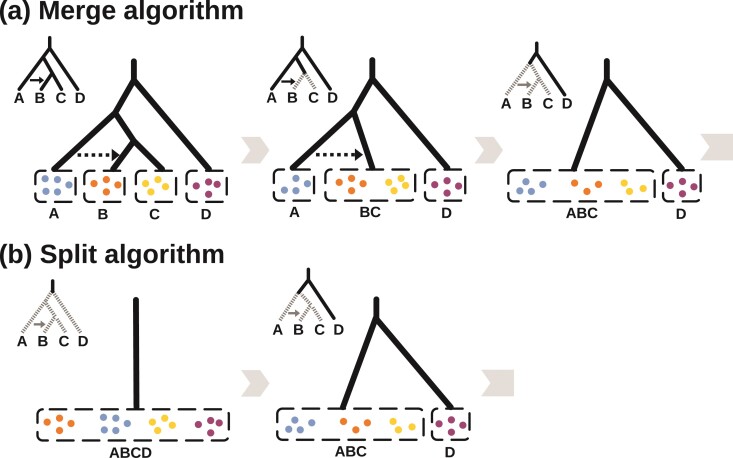
a) Hierarchical merge and b) hierarchical split algorithms applied to the same guide tree for 4 populations. The merge algorithm groups sister populations into 1 species only if gdi<0.2, while the split algorithm splits 1 species into 2 only if gdi>0.7. Because of the different cutoffs used, the merge algorithm may suggest more species than the split algorithm.

The hierarchical merge and split algorithms are illustrated in Figure 3. Both require the specification of a guide tree, possibly with gene flow. This may be based on the prior knowledge of the taxonomist or previous phylogenetic analyses of genetic or morphological data. We assume that specimens or samplesare already assigned to populations, which represent potentially distinct species. Our algorithms may group different populations into 1 species but never separate 1 population into multiple species. Prior knowledge may be used to specify migration events involving extant or extinct species/populations on the guide tree.

In the merge algorithm, we progressively group populations into the same species, starting from the tips of the tree and moving toward the root. A merge is accepted if either of the 2 gdi indexes (Equation (5)) is <0.2. The algorithm stops when no population pair can be merged (Fig. 3a).

In the hierarchical split algorithm, we start from the model of 1 species and progressively split each species into distinct species, starting from the root and moving toward the tips of the guide tree (Fig. 3b). The split is accepted if both gdi indexes (Equation (5)) are >0.5 and at least one is >0.7. The algorithm stops when no species can be split (Fig. 3b).

The merge and split algorithms are implemented under both the MSC model with no gene flow (Rannala and Yang 2003; Flouri et al. 2018) and the MSC-M model with migration (Flouri et al. 2023). Under the MSC-M model, we retain the migration event in the merge algorithm when at least 1 of the 2 merged populations is involved in migration with a third species. For example, in the guide tree of Figure 3a, there is migration from A to B. When B and C are merged into 1 species/population (BC), we retain the migration event (now from population A to population BC). When A and BC are later merged, the now intra-population migration event is removed.

In analysis of any dataset both the merge and split algorithms should be applied. We note that the merge and split algorithms may produce different results, mainly because of the different cutoffs (0.2 versus 0.7) and the large interval of indecision (with 0.2<gdi<0.7), not because of the different algorithms (merge versus split). Under the model of no gene flow and if the gdi for each internal node is smaller than that for its mother node, the merge and split algorithms using the same cut-off will arrive at the same model of delimitation and phylogeny. Thus, one could run the merge (or split) algorithm alone, but twice, using the 2 cutoffs (0.2 and 0.7), and obtain the same 2 sets of results as our merge and split algorithms. It is also possible to use the cutoff 0.7 for merge and 0.2 for split, in which case the merge algorithm may delimit fewer species than split (an example is shown in Supplementary Table S2). In our current approach, the merge algorithm may infer more species than the split algorithm and the approach has a computational advantage as it may involve fewer bpp runs. Of course, this reasoning serves as a rough guide only, as it may not apply when there is gene flow in the model and when a mother node has a smaller gdi than a daughter node.

### Computation of gdi Given Model Parameters

Given the parameters in the MSC or MSC-M models, we use different methods to calculate gdi, depending on the presence and types of migration events involving the focal populations A and B. We consider 3 cases: (a) no gene flow into A or B, (b) gene flow between A and B but not from any other populations, and (c) gene flow from other populations into at least one of A and B.

(**a**) In case of no gene flow into A or B, $gdi_{\text{J}}$ and $gdi_{\text{K}}$ are equivalent and Equation (4) simplifies to Equation (2), which is used in the calculation. Note that gene flow from populations A and B into a third population does not affect our calculation of gdi for A and B or our assessment of the species status of A and B.

(**b**) If there is migration between A and B but no gene flow from any other population into A or B, we use the Markov chain theory developed in the structured coalescent to calculate $gdi_{\text{K}}=P\left(G_{1a}\right)$analytically.

Given 2 populations (A and B) with gene flow, the process of coalescent and migration when one traces the genealogical history of the sample (of sequences $a_{1},a_{2},b$) backwards in time can be described by a Markov chain, in which the states are specified by the number of sequences remaining in the sample and the population IDs (A and B) and sequence IDs ($a_{1},a_{2},b$) (Supplementary Table S1) (Hobolth et al. 2011; Zhu and Yang 2012; Jiao and Yang 2021). The initial state is $A_{a_{1}}A_{a_{2}}B_{b}$, with 3 sequences $a_{1},a_{2},b$ in populations A, A, and B, respectively. This is also written “AAB”. State $A_{a_{1}a_{2}}B_{b}$, abbreviated “$AB_{b}$,” means that sequences $a_{1}$ and $a_{2}$ have already coalesced so that 2 sequences remain in the sample, with the ancestor of $a_{1}$ and $a_{2}$ in A while b is in B. Finally state A|B is an artificial absorbing state, in which all 3 sequences have coalesced with the sole ancestral sequence in either A or B. There are 21 states in the Markov chain, with the transition rate (generator) matrix $Q=\left\{q_{ij}\right\}$ given in Supplementary Table S1 (Leaché et al. 2019).

The transition probability matrix over time t is then $P\left(t\right)=\left\{p_{ij}\left(t\right)\right\}=e^{Qt}$, where $p_{ij}\left(t\right)$ is the probability that the Markov chain is in state j at time t (in the past) given that it is in state i at time 0 (the present time). Suppose Q has the spectral decomposition


$$q_{ij}=\sum_{k=1}^{21}u_{ik}v_{kj}\lambda_{k},$$ (6)

where $0=\lambda_{1}>\lambda_{2}\geq \cdots \geq \lambda_{21}$ are the eigenvalues of Q, and columns in $U=\left\{u_{ij}\right\}$ are the corresponding right eigenvectors, with $V=\left\{v_{ij}\right\}=U^{-1}$. Then


$$p_{ij}\left(t\right)=\sum_{k=1}^{21}u_{ik}v_{kj}\,e^{\lambda_{k}t}.$$ (7)

Gene tree $G_{1a}$ arises if sequences $a_{1}$ and $a_{2}$ coalesce first and before τ (as in the blue gene tree of Fig. 2b), and the coalescence can occur in either populations A or B. The coalescent time t has thedensity


$$\begin{array}{cc} f\left(t\right) & =\left[p_{AAB,AAA}\left(t\right)+p_{AAB,AAB}\left(t\right)\right]\frac{2}{\theta_{A}}\\ & +\left[p_{AAB,BBA}\left(t\right)+p_{AAB,BBB}\left(t\right)\right]\frac{2}{\theta_{B}},t<\tau . \end{array}$$ (8)

The 2 terms in the sum correspond to the coalescence occurring in A and B, respectively. For example, the first term is the probability that both $a_{1}$ and $a_{2}$ are in A right before time t (corresponding to states AAA or AAB), $p_{AAB,AAA}\left(t\right)+p_{AAB,AAB}\left(t\right)$, times the coalescent rate $2/\theta_{A}$. Similarly the second term is the probability density that $a_{1}$ and $a_{2}$ coalesce at time t in B, given by the probability that $a_{1}$ and $a_{2}$ are in B right before time t times the coalescent rate $2/\theta_{B}$.

By averaging over the distribution of t, we have


$$gdi_{\text{K}}=P\left(G_{1a}\right)=\int_{0}^{\tau }f\left(t\right)\,dt.$$ (9)

To calculate the integral in Equation (9), note that from Equation (7),


$$\int_{0}^{\tau }p_{ij}\left(t\right)\,dt=u_{i1}v_{1j}\tau +\sum_{k=2}^{21}u_{ik}v_{kj}\frac{e^{\lambda_{k}\tau }-1}{\lambda_{k}}.$$ (10)

Furthermore, the probability for gene tree $G_{1b}$ (Fig. 1) is


$$P\left(G_{1b}\right)=p_{AAB,s_{3}}\left(\tau \right)\times \frac{1}{3},$$ (11)

where $s_{3}=\{AAA$, AAB, ABA, ABB, BAA, BAB, BBA, BBB} is the set of states with 3 sequences. For $G_{1b}$ to occur, there must be no coalescence in the time interval (0,τ) and all 3 sequences must reach time τ, and then the 3 sequences coalesce in random order. Thus


$$P\left(G_{1}\right)=P\left(G_{1a}\right)+P\left(G_{1b}\right),$$ (12)

from which $gdi_{\text{J}}$ (Equation (3)) can be calculated.

(**c**) When populations A or B are recipients of gene flow from other populations, analytical calculation of the gdi becomes complicated. It is simpler to simulate a large number ($10^{6}$ or $10^{7}$, say) of gene trees under the migration model. Note that other populations on the guide tree than the focal populations A and B may contribute migrants into A or B. Parameters in the MSC-M model (τs, θs, and M) involving all those populations are estimated by bpp from the data. Gene trees for only 3 sequences ($a_{1},a_{2},b$) are then simulated, with no samples taken from other populations (see Supplementary Fig. S1 for an example control file for such simulation). The $gdi_{\text{K}}$ is simply the proportion for gene tree $G_{1a}$, that is, $G_{1}$ with $t_{1}<\tau_{AB}$, among simulated gene trees (Fig. 1):


$$gdi_{\text{K}}=P\left(G_{1a}\right)\approx \frac{\text{\# of gene tree }G_{1a}}{R},$$ (13)

where R is the number of replicate loci or gene trees.

Note that in cases (a) and (b), one could also use simulation to calculate gdi, but the analytical calculation is more accurate and computationally moreefficient.

### Uncertainty in gdi

The above describes the calculation of gdi given the parameters in the model (either with or without gene flow). In real data analysis, parameters are estimated from the sequence data and involve uncertainties due to the finite nature of data. A simple approach is to use the posterior means of parameters to calculate gdi. A more proper approach is to treat gdi as a function of the parameters and generate its posterior distribution and to use the posterior mean of gdi in the algorithm. The 2 approaches should be very similar if the dataset is informative and the parameters are wellestimated.

Let $\left\{\Theta^{\left(i\right)}\right\}$ be the parameter values sampled from the MCMC (with the definition of Θ depending on the model). Then for each i, calculate $gdi^{\left(i\right)}=gdi\left(\Theta^{\left(i\right)}\right)$ using 1 of the 3 approaches discussed in the last subsection. These $gdi^{\left(i\right)}$ values constitute a sample from the posterior distribution and can be used to calculatethe posterior mean, and can also be sorted to generate the 95% equal-tail credible interval (CI). The 95% highest probability density (HPD) CI can be calculated by sliding the 95% equal-tail CI to the left and to the right until the induced interval cannot be made shorter, relying on the fact that the HPD interval is the shortest (Chen and Shao 1999; see Fig. 7.14 in Yang 2014). We implemented a simple algorithm under the assumption that the HPD CI consists of 1 interval rather than several non-overlapping subintervals. Note that for the MSC-M model involving gene flow from other populations into A or B (case **c**), this procedure involves simulating many gene trees for each set of parameters $\Theta^{\left(i\right)}$. Thus, we may “thin” the MCMC sample to use only 1000 sets of parameter values.

### Implementation of hhsd

Our pipeline creates control files and Imap files to drive the analyses using bpp (an Imap file maps individual samples to species/populations under the specified species-delimitation hypothesis). It then examines the bpp output to calculate gdi to attempt to merge populations or split species. If any merge (or split) occurs the species tree is modified and new bpp control and Imap files are generated for the next iteration of the hierarchical algorithm. The pipeline is itself driven by a control file. Many of the control variables are the same as used in bpp, and the same syntax is used between the 2 programs as much as possible.

**Figure 4:**
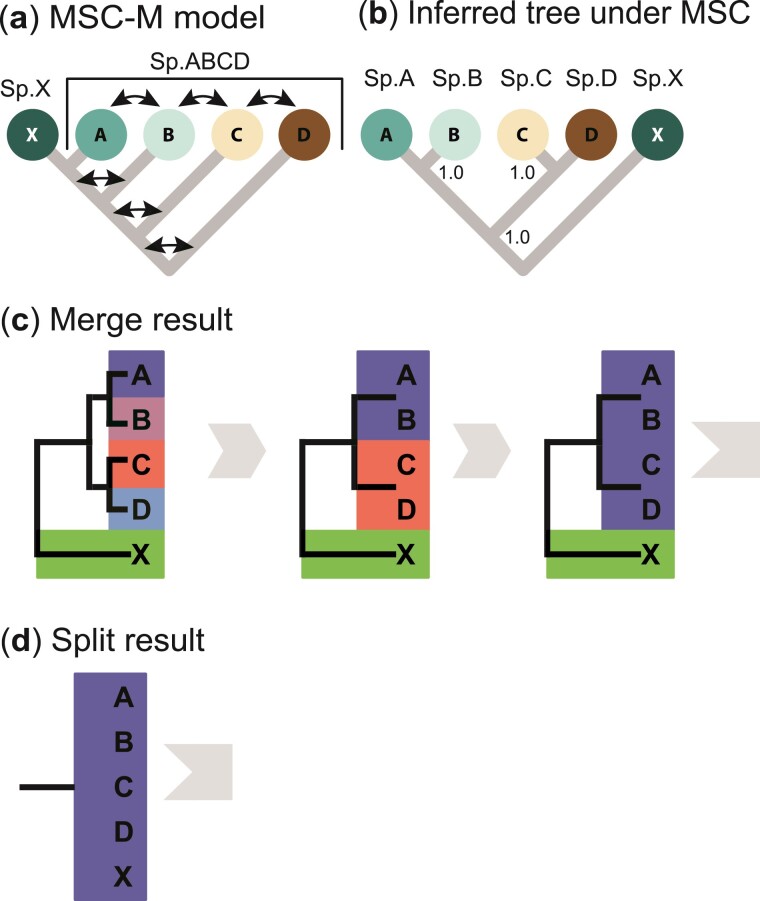
a) An isolation-by-distance model in which populations A,B,C, and D represent geographical populations of the same species, while population X is a distinct species that split from and remains in complete isolation with population A. The model is used to simulate multilocus sequence data. The parameters used are $\tau_{XABCD}=0.04$, $\tau_{XABC}=0.03$, $\tau_{XAB}=0.02$, and $\tau_{XA}=0.01$ for divergence times, and θ=0.01 for all populations, with M=Nm=2 between any 2 adjacent populations of the species ABCD. Redrawn after Leaché et al. (2019, Fig. 5). b) Incorrect species delimitation and phylogeny produced in Bayesian model selection using bpp under the MSC model assuming no gene flow, with every node receiving 100% posterior support. c) Output from the hhsd pipeline applying the merge algorithm under the MSC model to the simulated data (see Fig. 5 for the control file). The species tree of panel b) is used as the guide tree (initial delimitation). A merge is accepted if either $gdi_{A}$ or $gdi_{B}$ is <0.2. The algorithm recognizes 2 species: X and ABCD. d) Output from the split algorithm. A split is accepted if both $gdi_{A}$ and $gdi_{B}$ are >0.5 and at least one of them is >0.7. The algorithm infers 1 species (XABCD). The same data were also analyzed under the MSC-M model; see Supplementary Table S3 and text.

**Figure 5:**
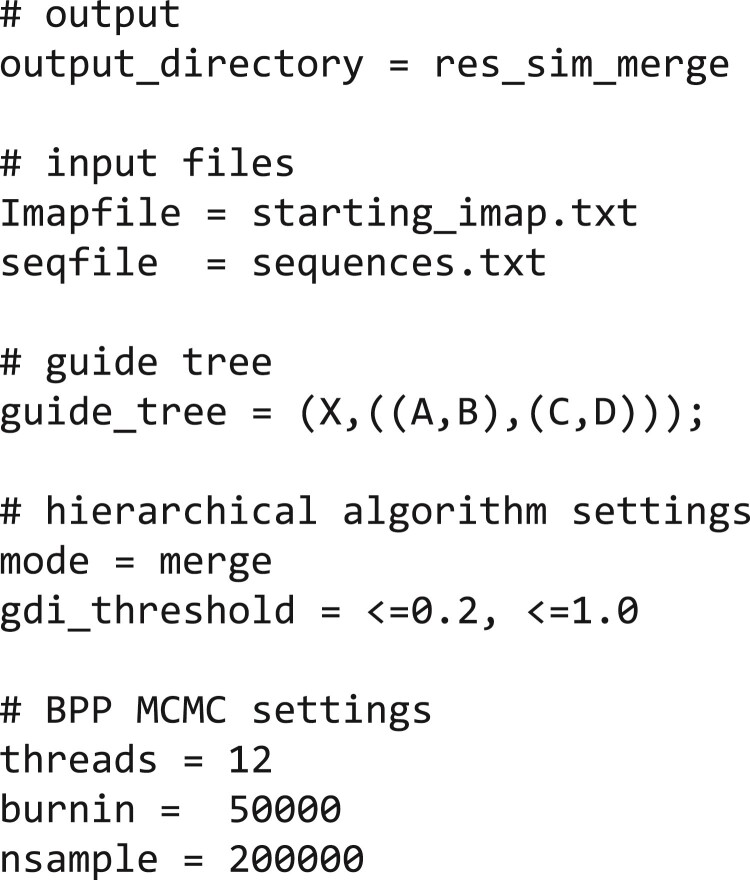
Control file (simulated_merge_analysis.txt) for hhsd merge analysis of the data simulated under the model of Figure 4a. The control variables are as follows: output_directory specifies the output directory in which result files will be written; seqfile is the sequence alignment file in phylip format; Imapfile specifies the assignment of individuals to populations; guide_tree is a Newick representation of the guide tree; and mode specifies the algorithm (merge or split). GDI_threshold specifies the gdi value below which 2 populations are merged into 1 species. threads specifies the number of CPU threads used to run bpp, while burnin, sampfreq, and nsample specify the MCMC settings for running bpp. Run hhsd using the command hhsd --cfile simulated_merge_analysis.txt

Here, we illustrate our pipeline through an analysis of a multilocus sequence dataset simulated under the isolation-by-distance model of Figure 4a (Leaché et al. 2019). The hhsd control file is shown in Figure 5. There are 5 populations, with A,B,C, D representing populations of a species with a wide geographic distribution, while X is a new species that split off from population A. There is extensive gene flow between any 2 neighbouring populations of species ABCD, with migration rate M=Nm=2 immigrants per generation, whereas there is no gene flow involving X (Fig. 4a). The data consisted of L=2000 loci, with S=4 sequences per species per locus, and 500 sites in the sequence.

The guide tree of Figure 4b, which is the starting delimitation for the merge algorithm, was generated using species tree estimation under the MSC model with no gene flow (i.e., the A01 analysis of Yang 2015). A manual run of the procedure is recorded in Supplementary Table S2 (using the cutoff gdi<0.2). The hhsd pipeline provides feedbacks about the current species delimitation and the decisions made during each iteration of the algorithm (Fig. 4c and Supplementary Fig. S2). In the first iteration, attempt was made to merge populations A and B, and C and D. As gdi<0.2 for each pair, both merges were accepted. In the second iteration, a merge between AB and CD was attempted, and again this was accepted. In the third iteration, a merge between the pair ABCD and X was attempted. As gdi>0.2, the merge was rejected. The final delimitation had 2 species, ABCD and X.

### Behavior of the gdi Under Models of Gene Flow

The pattern of gene flow under the MSC-M model may be very complex in terms of the number of gene-flow events, the lineages involved, and the directions and rates of gene flow. Gene flow is also known to exert profound impacts on the genealogical history of sequences sampled from modern species (Leaché et al. 2014; Long and Kubatko 2018; Jiao et al. 2020; Jiao and Yang 2021). Here, we characterize the behavior of the gdi under a few simple scenarios of gene flow, and leave it to the future to explore more complexmodels.

#### Case (a) Symmetrical migration model for 2 populations

**Figure 6:**
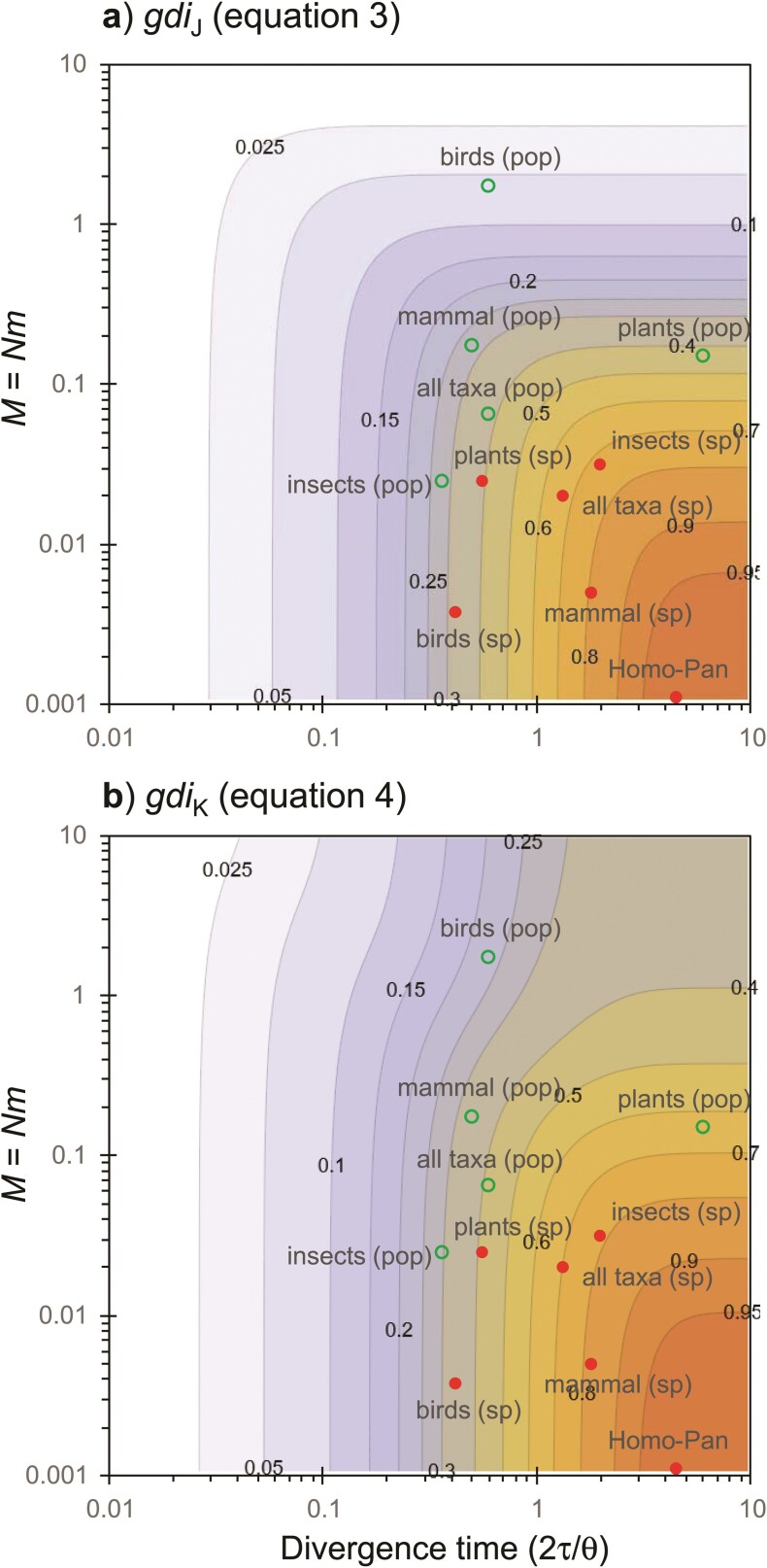
[Case a] a) $gdi_{\text{J}}$ and b) $gdi_{\text{K}}$ plotted against the population split time in coalescent units (2τ/θ=T/2N) and the population migration rate (M=Nm) under the symmetrical migration model for 2 populations, with $\theta_{A}=\theta_{B}=\theta$ and $M_{AB}=M_{BA}=M$ (Fig. 2a). The cut-offs at 0.2 and 0.7 are indicated by red contour lines. The green circles (between populations) and red dots (between species) represent median values of empirical estimates from major taxonomic groups (mammals, birds, insects, and plants) from the meta-analysis of Jackson et al. (2017, Fig. 6), based on data compiled by Pinho and Hey (2010, Supplementary Table S1). Panel a) is a transformation of $P\left(G_{1}\right)$ of Leaché et al. (2019, Fig. 3) using Equation (2). Under this symmetrical MSC-M model, there is no difference between $gdi_{A}$ and $gdi_{B}$ of Equation (5) and also $gdi_{\text{J}}$ is always >0.

The symmetrical migration model for 2 populations, with $N_{A}=N_{B}=N$ and $M_{AB}=M_{BA}=M$ (Fig. 2a), has been used by Jackson et al. (2017) and Leaché et al. (2019) to calculate $gdi_{\text{J}}$ (Equation (3)). Under this model, both $gdi_{\text{J}}$ and $gdi_{\text{K}}$ are functions of 2 parameters: 2τ/θ=T/(2N) and M. In Figure 6 we plot $gdi_{\text{J}}$ and $gdi_{\text{K}}$ for a range of values for those 2 parameters. Overall large population split time and low migration rate correspond to high *gdi* values and the species status of the 2 populations.

The 2 definitions ($gdi_{\text{J}}$ and $gdi_{\text{K}}$) are very similar in the whole parameter space except for the Northeast corner where both the migration rate and population split time are large. In such a scenario, the 2 populations should be considered 1 species according to $gdi_{\text{J}}$ (Fig. 6a), while the species status is ambiguous according to $gdi_{\text{K}}$ (Fig. 6b). The 2 indexes represent different biological interpretations of the same population divergence history, akin to 2 species concepts. We leave it to the future to evaluate which of them better matches the experience and expectation of taxonomists.

#### Case (b) Asymmetrical gene flow between 2 populations

Next, we consider an MSC-M model of unidirectional gene flow for 2 populations, with $M_{BA}>0$ and $M_{AB}=0$. This is a special case of the general model of Figure 2a considered in the Theory section and is analytically tractable. To track the history of sequences $a_{1},a_{2},b$ up to the split time τ, we use the generator matrix $Q^{\left(1\right)}$ of Jiao and Yang (2021):

**Table:** 

	AAB	ABB	BAB	BBB	$AB_{b}$	$A_{a_{1}}B$	$A_{a_{2}}B$	$BB_{b}$	$B_{a_{1}}B$	$B_{a_{2}}B$	B
AAB	$-2\varpi -c_{A}$	ϖ	ϖ	0	$c_{A}$	0	0	0	0	0	0
ABB	0	$-\varpi -c_{B}$	0	ϖ	0	$c_{B}$	0	0	0	0	0
BAB	0	0	$-\varpi -c_{B}$	ϖ	0	0	$c_{B}$	0	0	0	0
BBB	0	0	0	$-3c_{B}$	0	0	0	$c_{B}$	$c_{B}$	$c_{B}$	0
$AB_{b}$	0	0	0	0	−ϖ	0	0	ϖ	0	0	0
$A_{a_{1}}B$	0	0	0	0	0	−ϖ	0	0	ϖ	0	0
$A_{a_{2}}B$	0	0	0	0	0	0	−ϖ	0	0	ϖ	0
$BB_{b}$	0	0	0	0	0	0	0	$-c_{B}$	0	0	$c_{B}$
$B_{a_{1}}B$	0	0	0	0	0	0	0	0	$-c_{B}$	0	$c_{B}$
$B_{a_{2}}B$	0	0	0	0	0	0	0	0	0	$-c_{B}$	$c_{B}$
B	0	0	0	0	0	0	0	0	0	0	0

where $\varpi =4M/\theta_{A}=m_{BA}/\mu$, $c_{A}=2/\theta_{A}$, and $c_{B}=2/\theta_{B}$.

Let $P\left(t\right)=\left\{p_{ij}\left(t\right)\right\}=e^{Qt}$. To derive $gdi_{\text{K}}=P\left(G_{1a}\right)$, let t<τ be the coalescent time for sequences $a_{1}$ and $a_{2}$. As in Equation (8), this has density


$$f\left(t\right)=p_{AAB,AAB}\left(t\right)\cdot c_{A}+p_{AAB,BBB}\left(t\right)\cdot c_{B},t<\tau ,$$ (14)

where the 2 terms represent coalescence in populations A and B, respectively. Then


$$\begin{array}{cc} P\left(G_{1a}\right) & =\int_{0}^{\tau }f\left(t\right)\,dt\\ & =\frac{4e_{1}\theta_{B}^{2}M^{2}}{3\left(M\theta_{B}-\theta_{A}\right)\left(3\theta_{A}-\theta_{B}-4M\theta_{B}\right)}\\ & +\frac{4e_{2}\theta_{A}\theta_{B}^{2}M^{2}}{\left(\theta_{A}-M\theta_{B}\right)\left(\theta_{A}+2M\theta_{B}\right)\left(\theta_{A}-2M\theta_{B}-\theta_{B}\right)}\\ & +\frac{3\theta_{A}+2M\left(4M+3\right)\theta_{B}}{3\left(1+4M\right)\left(\theta_{A}+2M\theta_{B}\right)}-\frac{e_{3}}{1+4M}\\ & -\frac{8e_{3}\theta_{A}\theta_{B}M^{2}}{\left(\theta_{A}-\theta_{B}-2M\theta_{B}\right)\left(3\theta_{A}-\theta_{B}-4M\theta_{B}\right)\left(1+4M\right)}, \end{array}$$ (15)

where $e_{1}=\exp\left\{-6\tau /\theta_{B}\right\}$, $e_{2}=\exp\left\{-4M\tau /\theta_{A}-2\tau /\theta_{B}\right\}$ and $e_{3}=\exp\left\{-2\left(1+4M\right)\tau /\theta_{A}\right\}$.

Let $s_{3}=\left\{AAB,ABB,BAB,BBB\right\}$ be the set of states with 3 sequences. We have $P\left(G_{1b}\right)=p_{AAB,s_{3}}\left(\tau \right)\cdot 1/3$, and


$$\begin{array}{c} P\left(G_{1}\right)=P\left(G_{1a}\right)+P\left(G_{1b}\right)=\\ \frac{4\theta_{A}\theta_{B}e_{3}e_{4}\left(1+4M\right)M-\theta_{A}\theta_{B}\left(8M^{2}-3\right)-\theta_{A}\theta_{B}e_{3}\left(8M^{2}+2\right)}{3\left(1+4M\right)\left(\theta_{A}+2\theta_{B}M\right)\left(\theta_{B}+2\theta_{B}M-\theta_{A}\right)}\\ +\frac{\left(2e_{3}-4Me_{3}-3\right)\theta_{A}^{2}+2\theta_{B}^{2}M\left(2M+1\right)\left(4M+3-2e_{3}\right)}{3\left(1+4M\right)\left(\theta_{A}+2\theta_{B}M\right)\left(\theta_{B}+2\theta_{B}M-\theta_{A}\right)}, \end{array}$$ (16)

where $e_{4}=\exp\left\{4M\tau /\theta_{A}+2\tau /\theta_{A}-2\tau /\theta_{B}\right\}$.

**Figure 7:**
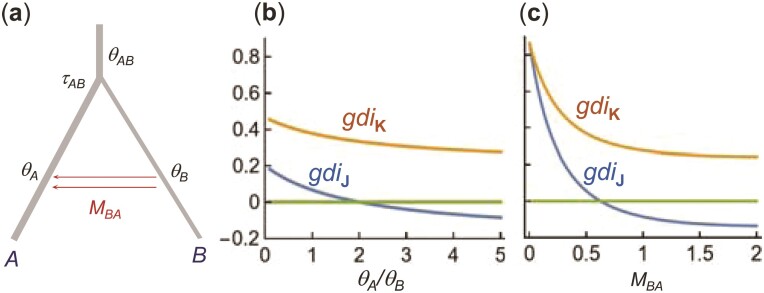
[Case b, aab data] a) An asymmetrical migration model for 2 populations (A,B) with migration from B to A. There are 5 parameters in the model, but $gdi_{\text{J}}$ and $gdi_{\text{K}}$ depend on only 3: $2\tau /\theta_{A}=T/\left(2N_{A}\right)$, $M=M_{BA}$, and $\theta_{A}/\theta_{B}=N_{A}/N_{B}$. b and c) $gdi_{\text{J}}$ and $gdi_{\text{K}}$ plotted against $N_{A}/N_{B}$ or $M_{BA}$, with $\tau =5\theta_{B}$ (the precise value of $\theta_{B}$ does not matter). In b), $M_{BA}=1$ is fixed, while in c), $\theta_{A}/\theta_{B}=5$ is fixed. When $N_{A}/N_{B}$ in b) or M in c) is large, the probability for the gene tree $G_{1}=\left(\left(a_{1},a_{2}\right),b\right)$ may be $<\frac{1}{3}$, so that $gdi_{\text{J}}<0$.

Both $gdi_{\text{J}}$ and $gdi_{\text{K}}$ are functions of 3 parameters: $2\tau /\theta_{A}=T/\left(2N_{A}\right)$, M, and $N_{A}/N_{B}$. Figure 7b,c shows that $gdi_{\text{J}}$ can be negative under this model. If population A has a much larger size than B, the 2 A sequences may not coalesce in A, and 1 of them may migrate into B (with time running backwards) and coalesce with sequence b, resulting in gene trees $G_{2}=\left(\left(a_{2},b\right),a_{1}\right)$ or $G_{3}=\left(\left(b,a_{1}\right),a_{2}\right)$. As a result, gene tree $G_{1}$ may be less probable than $G_{2}$ or $G_{3}$, creating an anomaly: 2 sequences from A are on average more distant from each other than either is from a sequence from B (Jiao and Yang 2021). See also Figure 2a in Jiao and Yang (2021).

**Figure 8:**
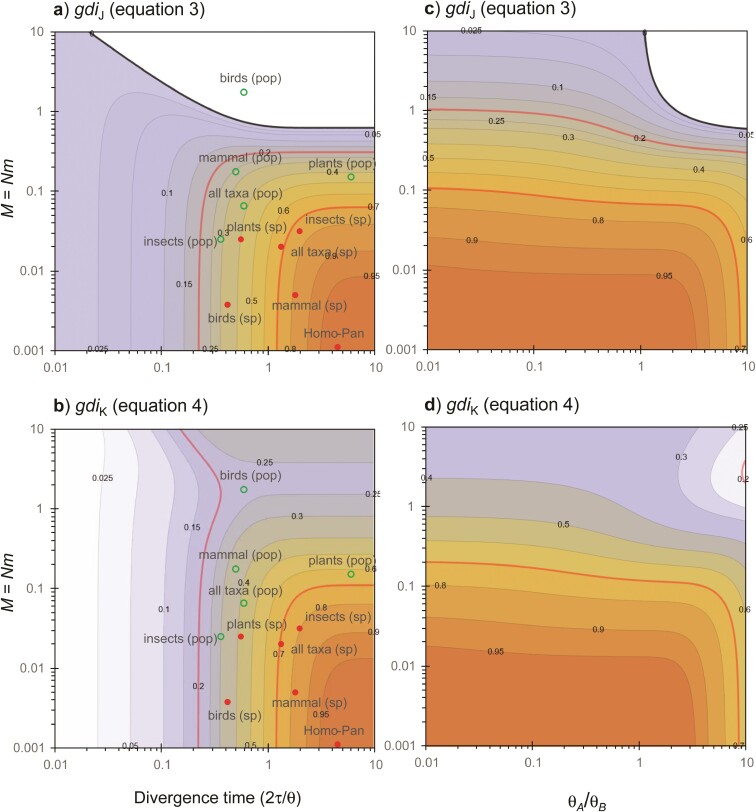
[Case b, aab data] a) $gdi_{\text{J}}$ and b) $gdi_{\text{K}}$ for sequences $a_{1},a_{2},b$ under the unidirectional migration model of Figure 7a, plotted against $M=M_{BA}$ and $2\tau /\theta_{A}$, with $\theta_{A}/\theta_{B}=5$ (the precise value of $\theta_{A}$ does not matter). c and d) Plots under the same model against M and $\theta_{A}/\theta_{B}$, with $\tau =5\theta_{B}$. In a) and c), $gdi_{\text{J}}<0$ in the white region outside the black contour line.

In Figure 8a,b, we plot $gdi_{\text{J}}$ and $gdi_{\text{K}}$ against M and $2\tau /\theta_{A}$, with $\theta_{A}/\theta_{B}=5$ fixed (the precise value of $\theta_{A}$ does not matter). In Figure 8c,d, we plot $gdi_{\text{J}}$ and $gdi_{\text{K}}$ against M and $\theta_{A}/\theta_{B}$, with $\tau =5\theta_{B}$ fixed (the precise value of $\theta_{B}$ does not matter). The 2 indexes behave in the same way except in the case of high migration rate and long divergence time, where $gdi_{\text{J}}$ lumps the 2 populations into 1 species, whereas $gdi_{\text{K}}$ is indecisive. This is the same pattern as under the symmetrical migration model of Figure 6.

We also considered the gdi with reference to population B, using sequences $a,b_{1},b_{2}$. We use the following generator matrix Q until the split time τ:

**Table:** 

	ABB	BBB	$A_{a}B$	$B_{a}B$	$BB_{b_{1}}$	$BB_{b_{2}}$	B
ABB	$-\varpi -c_{B}$	ϖ	$c_{B}$	0	0	0	0
BBB	0	$-3c_{B}$	0	$c_{B}$	$c_{B}$	$c_{B}$	0
$A_{a}B$	0	0	−ϖ	ϖ	0	0	0
$B_{a}B$	0	0	0	$-c_{B}$	0	0	$c_{B}$
$BB_{b_{1}}$	0	0	0	0	$-c_{B}$	0	$c_{B}$
$BB_{b_{2}}$	0	0	0	0	0	$-c_{B}$	$c_{B}$
B	0	0	0	0	0	0	0

where $\varpi =m_{BA}/\mu$ and $c_{B}=2/\theta_{B}$.

Let $P\left(t\right)=\left\{p_{ij}\left(t\right)\right\}=e^{Qt}$. The coalescent time t<τ for sequences $b_{1},b_{2}$ has density


$$f\left(t\right)=\left[p_{ABB,ABB}\left(t\right)+p_{ABB,BBB}\left(t\right)\right]\cdot c_{B},t<\tau ,$$ (17)

so that


$$\begin{array}{c} P\left(G_{1a}\right)=\int_{0}^{\tau }f\left(t\right)\,dt\\ =\frac{3\theta_{A}^{2}-2\theta_{B}^{2}M^{2}-\theta_{A}\theta_{B}M-3e_{1}e_{2}\theta_{A}^{2}+e_{1}\theta_{B}M\left(\theta_{A}+2\theta_{B}M\right)}{3\left(\theta_{A}-M\theta_{B}\right)\left(\theta_{A}+2M\theta_{B}\right)}, \end{array}$$ (18)

where $e_{1}=\exp\left\{-6\tau /\theta_{B}\right\}$ and $e_{2}=\exp\left\{-4M\tau /\theta_{A}+4\tau /\theta_{B}\right\}$.

As $P\left(G_{1b}\right)=\left[p_{ABB,ABB}\left(\tau \right)+p_{ABB,BBB}\left(\tau \right)\right]\cdot \frac{1}{3}$, we have


$$P\left(G_{1}\right)=P\left(G_{1a}\right)+P\left(G_{1b}\right)=\frac{\left(3-2e_{3}\right)\theta_{A}+2M\theta_{B}}{3\left(\theta_{A}+2M\theta_{B}\right)},$$ (19)

where $e_{3}=\exp\left\{-4M\tau /\theta_{A}-2\tau /\theta_{B}\right\}$.

**Figure 9:**
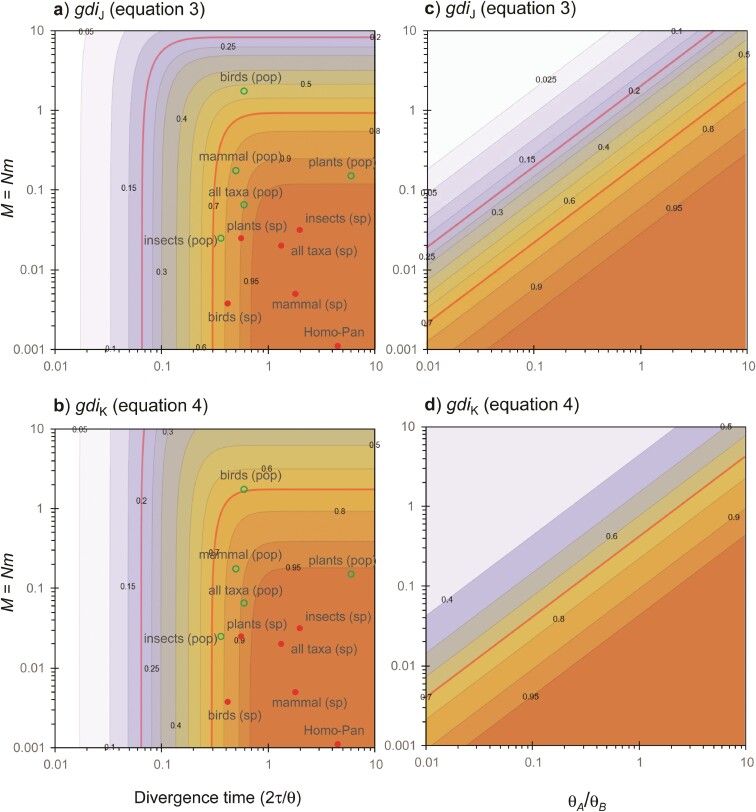
[Case b, for abb data] a) $gdi_{\text{J}}$ and b) $gdi_{\text{K}}$ for sequences $a,b_{1},b_{2}$ under the unidirectional migration model of Figure 7a, plotted against $M=M_{BA}$ and $2\tau /\theta_{A}$, with $\theta_{A}/\theta_{B}=5$. c and d) Plots under the same model against M and $\theta_{A}/\theta_{B}$, with $\tau =5\theta_{B}$. The model and parameter space are the same as in Figure 8 for aab data, and here $gdi_{\text{J}}$ is always positive.

Again both $gdi_{\text{J}}$ and $gdi_{\text{K}}$ are functions of 3 parameters: $2\tau /\theta_{A}=T/\left(2N_{A}\right)$, M, and $N_{A}/N_{B}$. In Figure 9a,b, we plot $gdi_{\text{J}}$ and $gdi_{\text{K}}$ for abb data (using sequences $a,b_{1},b_{2}$) over the same parameter space as in Figure 8. For abb data, the differencesbetween $gdi_{\text{J}}$ and $gdi_{\text{K}}$ are small (cf: Fig. 9a,b). However, there are large differences between $gdi_{A}$ and $gdi_{B}$ of Equation (5), reflecting the dramatic influence of the relative population sizes on the perceived species status of the populations (cf: Figs. 8c and 9c and Figs. 8d and 9d). For very small $N_{A}/N_{B}$, it is possible for $gdi_{A}>0.7$ and $gdi_{B}<0.2$. When population A has a much smaller size than population B, population A may appear to be a distinct species from B, while population B appears to be of the samespecies as A.

#### Case (c) Gene flow from a ghost species.—Markov chain at time

**Figure 10:**
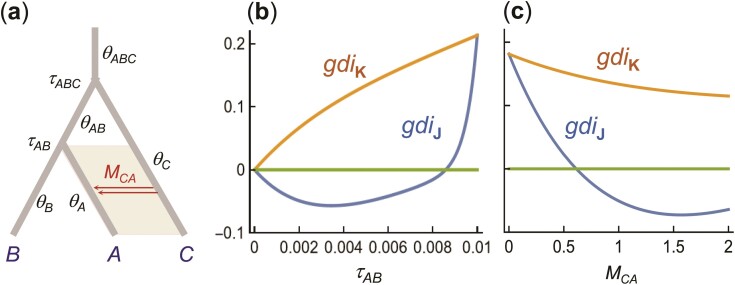
[Case c, aab data] a) An MSC-M model for 3 species (A,B,C) with migration from a ghost species C to A. In part of the parameter space, the probability for the gene tree $G_{1}=\left(\left(a_{1},a_{2}\right),b\right)$ is $<\frac{1}{3}$, with $gdi_{\text{J}}<0$ (Equation (3)). b and c) $gdi_{\text{J}}$ and $gdi_{\text{K}}$ plotted against $\tau_{AB}$ or $M_{CA}$ with $\tau_{ABC}=0.01$, $\theta_{A}=\theta_{C}=0.05$, and $\theta_{AB}=0.001$. In b), $M_{CA}=1$ is fixed, while in c), $\tau_{AB}=0.005$ is fixed.

In the model of Figure 10a, populations A and B have been in complete isolation since they diverged time $\tau_{AB}=\tau$ ago, but a more distant population C which diverged at time $\tau_{ABC}$ has been contributing migrants into population A at the rate of $M_{CA}=M$ migrants per generation. We sample sequences $a_{1}$ and $a_{2}$ from A and b from B, with no sample from C. The genealogical history of sequences $a_{1}$ and $a_{2}$ until time τ is described by a Markov chain with 4 states: AA,AC,CC,A|C, with the last being an absorbing state after the 2 sequences have coalesced. The generator matrix Q is

**Table:** 

	AA	AC	CC	A|C
AA	$-\left(2\varpi +c_{A}\right)$	2ϖ	0	$c_{A}$
AC	0	−ϖ	ϖ	0
CC	0	0	$-c_{C}$	$c_{C}$
A|C	0	0	0	0

where $\varpi \equiv \varpi_{CA}=m_{CA}/\mu$, $c_{A}=2/\theta_{A}$ and $c_{C}=2/\theta_{C}$. The eigenvalues of Q are $\lambda_{1}=0$, $\lambda_{2}=-c_{C}$, $\lambda_{3}=-c_{A}-2\varpi$, and $\lambda_{4}=-\varpi$.

Let $P\left(t\right)=\left\{p_{ij}\left(t\right)\right\}=e^{Qt}$. Given the initial state AA, the transition probabilities into the 4 states over time τ are


$$\begin{array}{cc} p_{11} & =e^{-\left(c_{A}+2\varpi \right)\tau },\\ p_{12} & =\frac{2\varpi }{c_{A}+\varpi }\left[e^{-\varpi \tau }-e^{-\left(c_{A}+2\varpi \right)\tau }\right],\\ p_{13} & =\\ & \frac{2\varpi^{2}\left[\left(c_{C}-\varpi \right)\,e^{-\left(c_{A}+2\varpi \right)\tau }-\left(c_{A}+\varpi \right)\,e^{-c_{C}\tau }+\left(c_{A}-c_{C}+2\varpi \right)\,e^{-\varpi \tau }\right]}{\left(c_{A}+\varpi \right)\left(c_{C}-\varpi \right)\left(c_{A}-c_{C}+2\varpi \right)},\\ p_{14} & =1-p_{11}-p_{12}-p_{13}\equiv P\left(G_{1a}\right). \end{array}$$ (20)

Note that the transition probability $p_{14}\left(\tau \right)$ is also $gdi_{\text{K}}=P\left(G_{1a}\right)$ of Equation (4). The probability for gene tree $G_{1}$ is given by averaging over the 4 possible states of the Markov chain at time τ,


$$\begin{array}{cc} P\left(G_{1}\right) & =p_{11}\times \frac{1}{3}+p_{12}\,e^{-2\Delta \tau /\theta_{AB}}\times \frac{1}{3}\\ & +p_{13}\left(1-\frac{2}{3}\,e^{-2\Delta \tau /\theta_{C}}\right)+p_{14}, \end{array}$$ 21

with $\Delta \tau =\tau_{ABC}-\tau_{AB}$, while $P\left(G_{2}\right)=P\left(G_{3}\right)=\left(1-P\left(G_{1}\right)\right)/2$. The first term in Equation (21) corresponds to state AA, with both $a_{1}$ and $a_{2}$ remaining in A at time τ (with probability $p_{11}$). Then all 3 sequences enter population AB and coalesce in random order, so that gene tree $G_{1}$ occurs with probability $\frac{1}{3}$. The second term corresponds to state AC at time τ, which means that one of $a_{1}$ and $a_{2}$ is in A with the other in C. If the sequence in A does not coalesce with b in the ancestral population AB, then gene tree $G_{1}$ will occur with probability $\frac{1}{3}$. The third term corresponds to state CC, with both $a_{1}$ and $a_{2}$ in C at time τ (with probability $p_{13}$). Then gene tree $G_{1}$ arises if $a_{1}$ and $a_{2}$ coalesce in C or in ABC. The fourth term, $p_{14}$, corresponds to state A|C, in which $a_{1}$ and $a_{2}$ have coalesced (in either A or C) before reaching τ so that the gene tree is $G_{1}$ (also $G_{1a}$) (Fig. 1).

The MSC-M model of Figure 10a involves 8 parameters, Θ= ($\tau_{ABC},\tau_{AB},\theta_{A},\theta_{B},\theta_{C},\theta_{AB},\theta_{ABC}$, and $M_{CA}$), but the gene-tree probability $P\left(G_{1}\right)$ is a function of 5: $2\tau /\theta_{A}=T_{AB}/\left(2N_{A}\right)$, $c_{A}/c_{C}=N_{C}/N_{A}$, $M_{CA}$, $\Delta \tau /\theta_{AB}$, and $\Delta \tau /\theta_{C}$. The new index $gdi_{\text{K}}=P\left(G_{1a}\right)$ is a function of the first 3 parameters.

As in the unidirectional migration model of Figure 7a, a similar anomaly arises under the model of Figure 10a with gene flow from a ghost species (Fig. 10b,c). For example, when the parameters are $\tau_{ABC}=0.01$, $\tau_{AB}=0.005$, $\theta_{A}=\theta_{C}=0.05$, $\theta_{AB}=0.001$, and $M_{CA}=1$, we have $P\left(G_{1}\right)=0.2995<\frac{1}{3}$ (Equation (21)), giving$gdi_{\text{J}}=-0.0508$. This is confirmed by simulation [see Supplementary Fig. S1 for the bpp control file for simulating gene trees in this case; parameters such as $\theta_{ABC}=0.01$ are needed to run the simulation program but do not affect $P\left(G_{1}\right)$]. As either of $a_{1}$ and $a_{2}$ may migrate into C (backwards in time), reducing the chance for $a_{1}$ and $a_{2}$ to coalesce in population A, gene tree $G_{1}$ may be less probable than $G_{2}$ or $G_{3}$, with $P\left(G_{1}\right)<P\left(G_{2}\right)=P\left(G_{3}\right)$.

**Figure 11:**
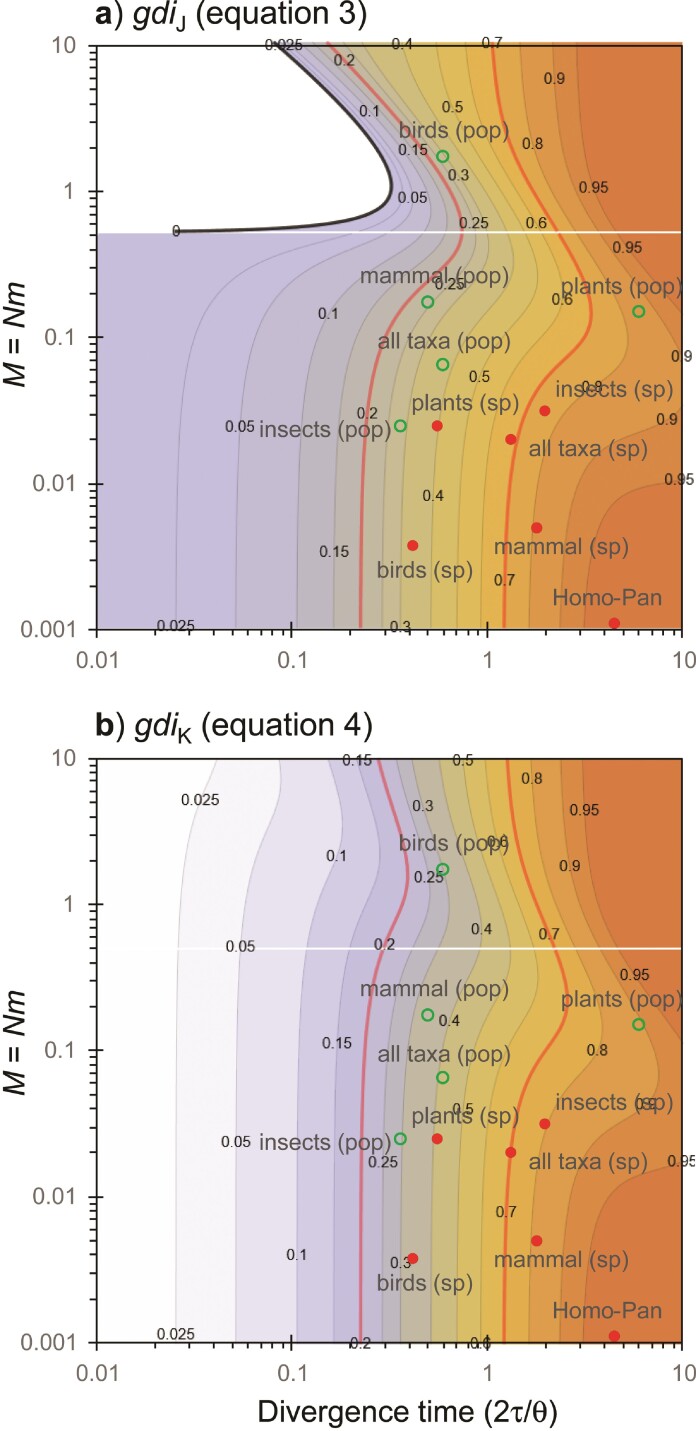
[Case c, aab data] a) $gdi_{\text{J}}$ and b) $gdi_{\text{K}}$ for sequences $a_{1},a_{2},b$ under the MSC-M model of Figure 10a with gene flow from a ghost species, plotted against $M=M_{CA}$ and 2τ/θ, with $\theta_{A}=\theta_{C}=\theta$, and $\Delta \tau =0.1\theta_{C}=5\theta_{AB}$. In a), $gdi_{\text{J}}<0$ in the white region outside the black contour line. We used the parameter values of Figure 10 in the calculation, but note that $gdi_{\text{J}}$ depends on only 5 parameters and $gdi_{\text{K}}$ depends on 3.

In Figure 11a,b we plot $gdi_{\text{J}}$ and $gdi_{\text{K}}$ against $M_{CA}$ and 2τ/θ, with other parameters fixed at the values of Figure 10. For those parameter values, $gdi_{\text{J}}$ and $gdi_{\text{K}}$ are very similar, although $gdi_{\text{J}}<0$ in part of the parameter space.

If we use instead abb data (with sequences $a,b_{1},b_{2}$), we have


$$P\left(G_{1}\right)=1-\frac{2}{3}\,e^{-2\tau /\theta_{B}},P\left(G_{1a}\right)=1-e^{-2\tau /\theta_{B}},$$

as in the case of no gene flow, and $gdi_{\text{J}}=gdi_{\text{K}}$.

There is thus a major asymmetry in the gdi index (Equation (5)) under this model: while $gdi_{A}$ for aab data depends on 5 or 3 parameters (for $gdi_{\text{J}}$ and $gdi_{\text{K}}$, respectively), $gdi_{B}$ for abb data depends on another unrelated parameter ($2\tau /\theta_{B}$). All possible scenarios are thus possible concerning $gdi_{A}$ versus $gdi_{B}$. For example, aab data may recognize A as a distinct species from B, while abb data may recognize B as of the same as A, or vice versa.

#### Case (d) Gene flow between non-sister lineages and paraphyletic species

Finally, we considered the species tree and MSC-M model of Figure 4a, in which populations A,B,C, and D represent 1 paraphyletic species with different geographical populations with excessive gene flow between them, while population X is a distinct species that split off from population A time $\tau_{XA}$ ago and has since been in complete isolation from population A or species ABCD. We conducted 2 analyses under the model. The first was an assessment of the gdi calculated for non-sister populations (such as A and B in Fig. 4a). The second was a re-analysis of the multilocus sequence data simulated under the model of Figure 4a, to explore the idea of merging non-sister lineages under the MSC-M model in the hierarchical merge algorithm to delimit paraphyletic species.

First, we explored the behavior of the gdi for non-sister populations. We simulated gene trees to calculate gdi for population pairs X–A, A–B, B–C, and C–D at different migration rates, with M=0,0.01,0.05,0.1,0.2,0.3,0.4,0.5,0.75,1,1.5, and 2. Other parameters are given in Figure 4a. For each M and each population pair, we simulated gene trees for 3 sequences (in either the aab or abb configuration) to calculate $gdi_{\text{J}}$ and $gdi_{\text{K}}$. For instance, for populations A and B and the aab configuration, we simulated $10^{6}$ gene trees for 3 sequences ($a_{1},a_{2},b$) under the MSC-M model for 5 populations of Figure 4a and calculated the proportions of gene tree $G_{1}$ as well as $G_{1a}$, that is, $G_{1}=\left(\left(a_{1},a_{2}\right),b\right)$ with the node age $t_{aa}<\tau_{XAB}$.

**Figure 12:**
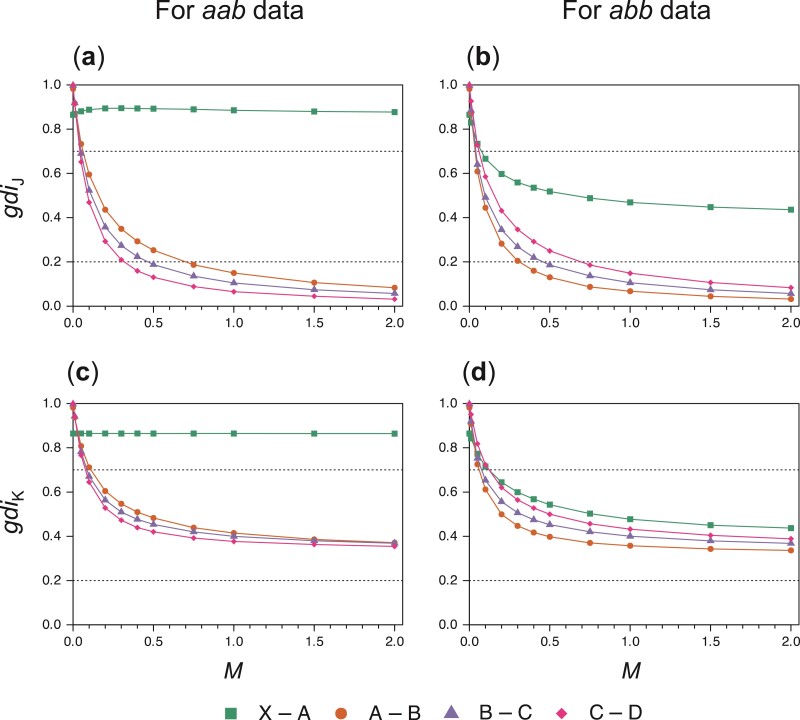
$gdi_{\text{J}}$ and $gdi_{\text{K}}$ for population pairs under the isolation-by-distance model of Figure 4a, plotted against the migration rate (M=Nm), estimated by simulating $10^{6}$ gene trees for 3 sequences. Parameters are fixed at the values in Figure 4a: $\tau_{XABCD}=0.04$, $\tau_{XABC}=0.03$, $\tau_{XAB}=0.02$, and $\tau_{XA}=0.01$, with θ=0.01 for all populations. Three sequences, in either the aab or abb configuration, are sampled per locus per population pair; in the case of populations A and B, they are either $a_{1},a_{2},b$, in which case the gene tree $G_{1}$ has the topology $\left(\left(a_{1},a_{2}\right),b\right)$; or $a,b_{1},b_{2}$, in which case $G_{1}$ is $\left(a,\left(b_{1},b_{2}\right)\right)$.

The results are shown in Figure 12. If there is little gene flow, with M=Nm≤0.05, all 5 populations (X,A,B,C,D) are considered distinct species using both indexes $gdi_{\text{J}}$ and $gdi_{\text{K}}$, and using both aab or abb data. However, at moderate levels of gene flow, the results depend on the index and the dataconfiguration.

Concerning the species status of X and A, the 2 indexes $gdi_{\text{J}}$ and $gdi_{\text{K}}$ are very similar, but there are substantial differences depending on whether one calculates gdi using xxa or xaa data. When one uses xxa, gdi>0.7 (Fig. 12a,c, X–A pair), and population X is judged to be a distinct species from A. However, with xaa data, gdi<0.7 when M>0.1 (Fig. 12b,d, X–A pair), and population A may not be considered a distinct species from X. The difference may be due to the fact that because of gene flow from population B, population A has a much larger effective population size than X.

Concerning the species status of populations A,B,C, and D, the data configuration (aab vs. abb) made little difference, but the 2 indexes $gdi_{\text{J}}$ and $gdi_{\text{K}}$ behaved differently. When M>0.5, $gdi_{\text{J}}$ assigned populations A,B,C, and D to the same species ($gdi_{\text{J}}<0.2$, Fig. 12a,b), while $gdi_{\text{K}}$ is indecisive ($0.2<gdi_{\text{K}}<0.7$, Fig. 12c,d). This appeared to be the same pattern as in the symmetrical migration model of case (**a**) (Fig. 6).

Second, we analyzed the XABCD dataset simulated under the MSC-M model of Figure 4a. Earlier these data were analyzed under the MSC model with no gene flow, using the guide tree of Figure 4b, which had a different topology from the true species tree of Figure 4a. With no gene flow in the model, $gdi_{\text{J}}$ and $gdi_{\text{K}}$ are equivalent, and both inferred either 1 species (XABCD) at the cutoff of gdi=0.7 or 2 species (X and ABCD) at the cutoff of gdi=0.2.

Here, we re-analyzed the same data under the MSC-M model, allowing the merge of non-sister populations as a strategy for delimiting paraphyletic species. We ignored the problem of inferring the MSC-M model with gene flow from genomic data (see Flouri et al. 2023 for discussions), and used the true MSC-M model of Figure 4a as the guide tree (or starting delimitation). In each iteration, we allow the merging of multiple pairs of sister lineages. If no sister pair can be merged, we consider non-sister pairs and allow the merge of only 1 non-sister pair (corresponding to the smallest gdi). After each merge, migration events between the merged populations are removed. Two cutoffs, 0.2 and 0.7, are used in the algorithm. This procedure is not yet automated in the hhsd pipeline, and instead we implemented it manually (Supplementary Table S3). $gdi_{\text{J}}$ supported 2 species (X and ABCD) at the cutoff gdi<0.2 or 1 species at the cutoff gdi<0.7. In contrast, $gdi_{\text{K}}$ identified 5 species at the cutoff gdi<0.2 or 1 species (XABCD) at the cutoff gdi<0.7. The results agreed well with the theoretical calculations of Figure 12.

## Results from Empirical Datasets

We analyzed 3 empirical datasets using the hhsd pipeline. In each case, the specific taxonomic group and associated delimitation problem will be introduced along with existing results.

### Species Delimitation of Giraffes (Genus *Giraffa*)

The taxonomic position and classification of giraffes (genus *Giraffa*) have been controversial for many years (Mitchell 2009). Previous studies using morphological characters and molecular data produced inconsistent results, delimiting from 1 to 6 species in the *Giraffa* genus. Currently, 9 geographical populations are recognized as subspecies: *camelopardalis*, *angolensis*, *antiquorum*, *giraffa*, *peralta*, *reticulata*, *rothschildi*, *thornicrofti*, and *tippelskirchi*. Most recently, Petzold and Hassanin (2020) compiled a multilocus dataset of 21 introns (average sequence length 808 bp), sampled from 66 individuals from the 9 subspecies, and conducted a number of population genetic and phylogenetic analyses. The authors suggested a delimitation with 3 species, although they noted that Bayesian model selection by bpp supported as many as 5 species.

**Figure 13:**
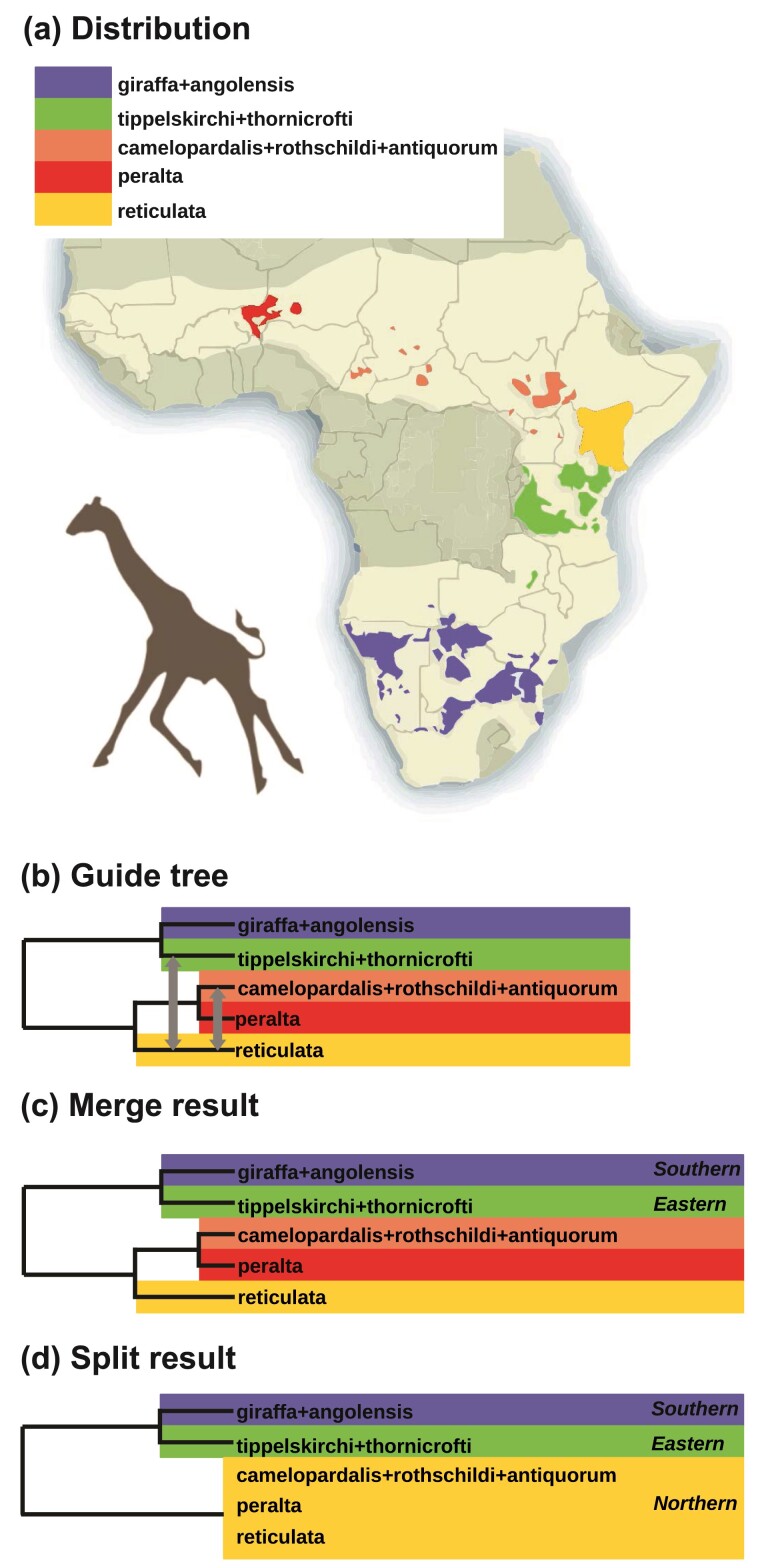
a) Geographical distributions of 5 putative species within *Giraffa*. The bright region on the map (modified from https://giraffeconservation.org/giraffe-species/) shows historical (ca. 1700) giraffe ranges. b) The guide tree for 5 populations of giraffes, with gray arrows indicating bidirectional migration events (Petzold and Hassanin 2020, Fig. 1). c) The merge algorithm supports 5 species, while d) the split algorithm supports 3.

We re-analyzed these data using our pipeline, using the 5-species phylogeny (Fig. 13b) as the guide tree, which was inferred using bpp by Petzold and Hassanin (2020). Based on phylogenetic analysis of mitochondrial haplotypes and identified hybrids (Fennessy et al. 2016; Petzold and Hassanin 2020), bidirectional migration was specified between *reticulata* and the *tippelskirchi+thornicrofti* lineage, and between *reticulata* and the *camelopardalis+rothschildi+antiquorum* lineage. Migration rates were assigned the gamma prior G(0.1, 10) with a mean of 0.1/10=0.01 migrant individuals per generation. Merge and split analyses were conducted with the animal-specific gdi thresholds of 0.3 and 0.7, as recommended by Jackson et al. (2017) (see Supplementary Fig. S3 for the control file). Each iteration of the algorithms took ∼2h using 8 threads on a server with Intel Xeon Gold 6154 CPU, with a total runtime of approximately 8 h.

The merge algorithm suggested 5 species, while the split algorithm suggested 3 (Fig. 13c,d). Both methods recognized the Eastern (*tippelskirchi* and *thornicrofti*) and Southern (*giraffa* and *angolensis*) populations in the guide tree as distinct species. The split algorithm lumped the 3 Northern populations into 1 species, while the merge algorithm recognized them as 3 distinct species.

Estimates of migration rates during the merge algorithm supported the hypothesized patterns of gene flow between reticulated giraffes and the neighbouring populations (Table 1). The highest migration rate was between the Northern populations from *cam.+rot.+ant.* to *reticulata*.

**Table 1:** Estimates (posterior means and 95% HPD CIs) of migration rates (M) between the 5 putative giraffe species in the guide tree of Figure 13b.

Donor	Recipient	M (95% HPD CI)
TipTho	reticulata	0.002 (0.000, 0.015)
reticulata	TipTho	0.002 (0.000, 0.009)
reticulata	CamRotAnt	0.027 (0.000, 0.129)
CamRotAnt	reticulata	0.123 (0.000, 0.328)

### Species Delimitation in Milksnakes (*Lampropeltis triangulum*)

**Figure 14:**
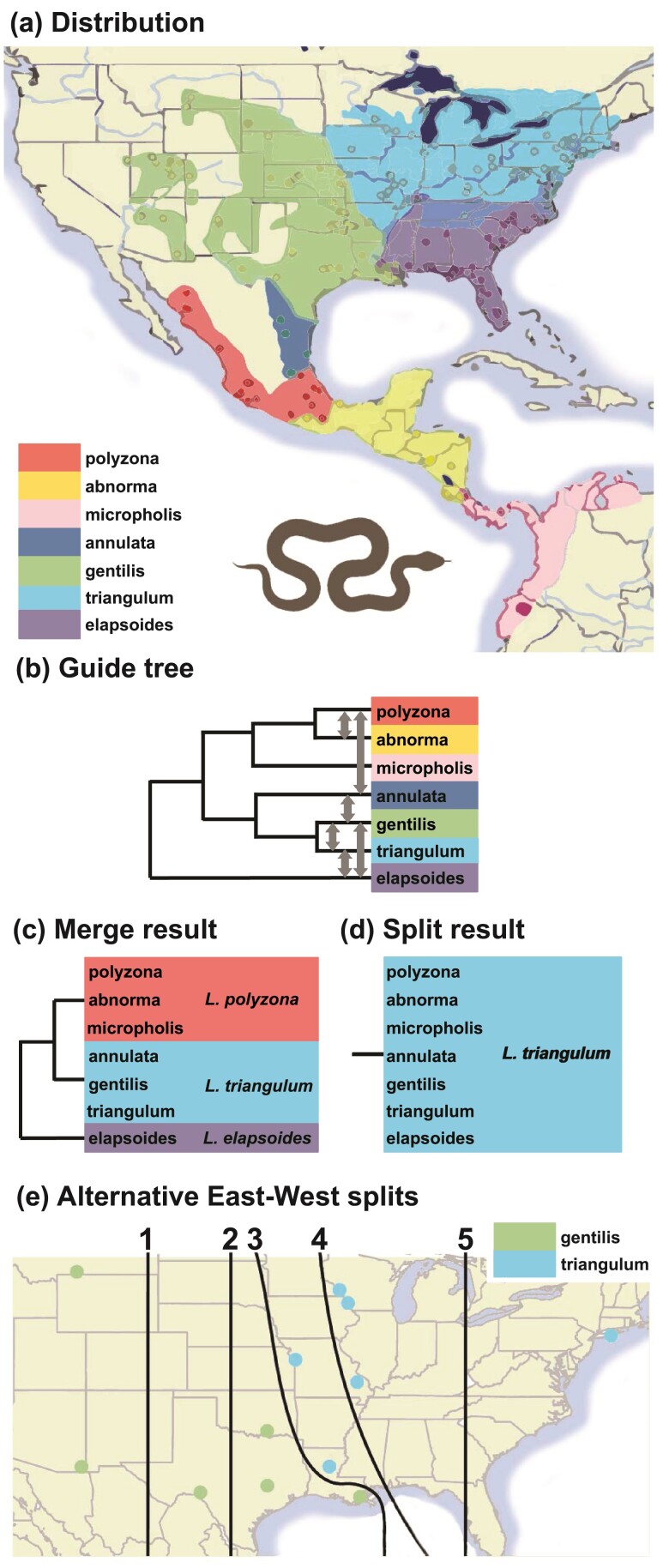
a) Geographic distribution of 7 milksnake subspecies (map based on and modified from Ruane et al. 2014, Fig. 1d). b) The guide tree with bidirectional migration events indicated by gray arrows. c and d) Inferred delimitation hypotheses by the merge and split algorithms. e) Alternative delimitation hypotheses tested by Chambers and Hillis (2020), each of which splits the *gentilis* and *triangulum* samples at an arbitrary West-East divide line. The hhsd merge algorithm grouped the 2 populations in each hypothesis into a single species.

The American milksnake *Lampropeltis triangulum* is a New World snake with one of the widest known geographic distributions within the squamates. Seven subspecies are known: *abnorma*, *polyzona*, *micropholis*, *triangulum*, *gentilis*, *annulata*, and *elapsoides* (Fig. 14a). Ruane et al. (2014) analyzed 11 nuclear loci (average length 537 bp) for 164 individuals from the 7 subspecies using bpp model comparison and found evidence for 7 distinct species. Chambers and Hillis (2020) re-analyzed these data and suggested that several species hypothesized by Ruane et al. (2014) may represent arbitrary slices of continuous geographic clines. They instead suggested 2 delimitation hypotheses, with 3 and 1 species, respectively, as shown in Figure 14c, d.

We re-analyzed the data of Ruane et al. (2014) using our pipeline, using the guide tree for 7 populations of Chambers and Hillis (2020) (Fig. 14b). As the original analysis Ruane et al. (2014) found ongoing gene flow between geographically adjacent populations, we added bidirectional migration events in the guide tree (Fig. 14b). Merge and split algorithms were run using gdi thresholds of 0.3 and 0.7 (see Supplmentary Fig. S4 for the control file). Each iteration of the algorithm took ∼2.5 h using 8 threads on a server, with a total runtime of ∼12.5 h.

The merge algorithm suggested 3 species, grouping the subspecies *abnorma*, *polyzona*, and *micropholis* into 1 species, and *triangulum*, *gentilis*, and *annulata* into another species (Fig. 14c). This is the same delimitation as the 3-species hypothesis of Chambers and Hillis (2020). The split analysis supported only 1 species (Fig. 14d). Migration rates between the adjacent subspecies/populations during the merge analysis suggested ongoing genetic exchange between some of the subspecies pairs, in particular, between *L. annulata* and *L. gentilis*, and between *L. abnorma* and *L. polyzona*(Table 2).

**Table 2:** Estimates of migration rates (M) between 7 milksnake populations during the merge algorithm (Figure 14).

It.	Donor	Recipient	M (95% HPD CI)
1	elapsoides	gentilis	0.003 (0.000, 0.019)
	gentilis	elapsoides	0.010 (0.000, 0.055)
	elapsoides	triangulum	0.009 (0.000, 0.052)
	triangulum	elapsoides	0.055 (0.000, 0.152)
	annulata	polyzona	0.002 (0.000, 0.012)
	polyzona	annulata	0.003 (0.000, 0.016)
	annulata	gentilis	0.162 (0.000, 0.331)
	gentilis	annulata	0.053 (0.000, 0.220)
	polyzona	abnorma	0.044 (0.000, 0.189)
	abnorma	polyzona	0.127 (0.000, 0.293)
	gentilis	triangulum	0.011 (0.000, 0.070)
	triangulum	gentilis	0.050 (0.000, 0.267)
2	elapsoides	GenTri	0.008 (0.000, 0.049)
	GenTri	elapsoides	0.067 (0.000, 0.142)
	annulata	PolAbn	0.002 (0.000, 0.010)
	PolAbn	annulata	0.003 (0.000, 0.020)
	annulata	GenTri	0.081 (0.000, 0.276)
	GenTri	annulata	0.073 (0.000, 0.181)
3	elapsoides	AnnGenTri	0.016 (0.000, 0.085)
	AnnGenTri	elapsoides	0.102 (0.028, 0.184)
	MicPolAbn	AnnGenTri	0.006 (0.000, 0.034)
	AnnGenTri	MicPolAbn	0.080 (0.028, 0.135)

Chambers and Hillis (2020) also applied an arbitrary West-East divide to split the *gentilis* and *triangulum* populations into 2 species, generating 5 arbitrary delimitation hypotheses (each with 2 species) (Fig. 14e). They found that all 5 delimitation hypotheses were supported by Bayesian model selection using bpp, even though they are arbitrary. We used our pipeline to re-analyze the data, using the merge algorithm with the same settings as above. The data consisted of only the 38 individuals from *gentilis*, *triangulum*, and *annulata* populations. The same guide tree for the 3 populations was used, but each hypothesis was represented by constructing an Imap file to map the individual samples to the 3 populations (see Supplementary Figs. S5 and S6 for the control file and command-line scripts). Bidirectional migration between *gentilis* and *triangulum* was allowed in the guide tree. Each iteration of the algorithm took ∼1.5 h on a server using 8 threads, with a total runtimeof ∼15 h.

Under each of the 5 delimitation hypotheses, the hhsd merge algorithm grouped the 2 subspecies *gentilis* and *triangulum* into a single species.

### Introgression and Species Delimitation in the Longear Sunfish (*Lepomis megalotis*)

**Figure 15:**
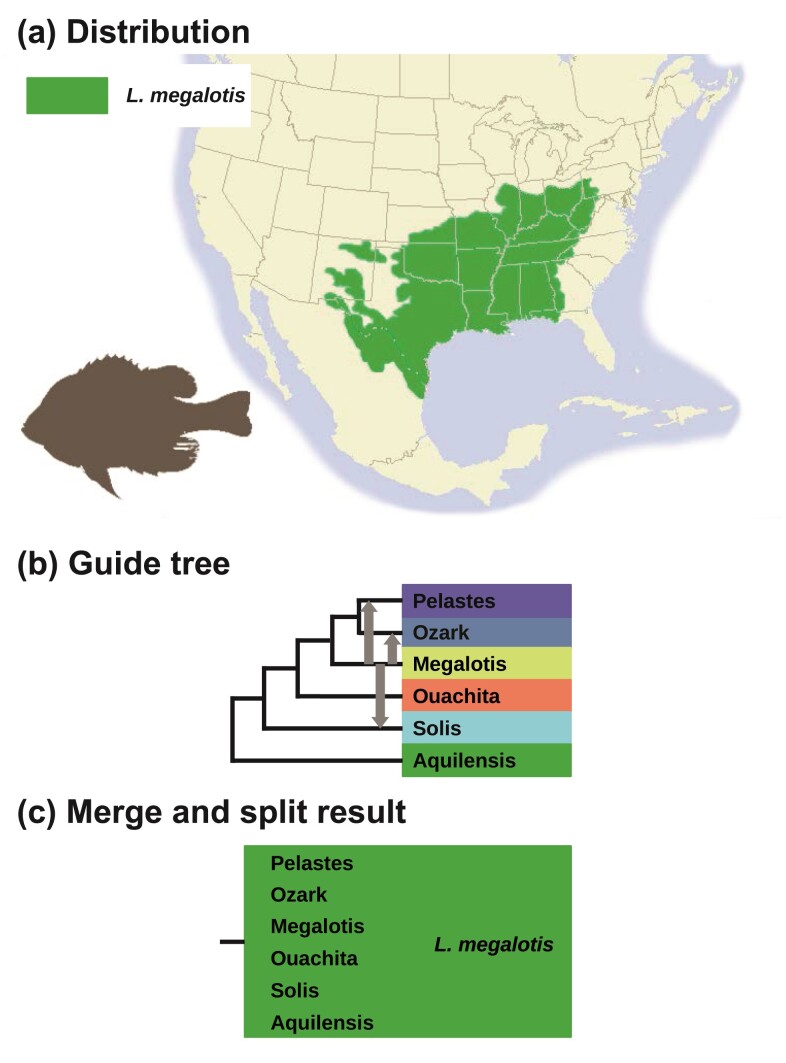
a) Geographic distribution of longear sunfish (*Lepomis megalotis*) (map based on http://www.roughfish.com/content/longear-sunfish). b) The guide tree, with 3 migration events (from *L. megalotis* to *L. pelastes*, *L. solis*, and *L. ozark*) indicated by gray arrows. c) Both merge and split algorithms support a single species.

The longear sunfish (*Lepomis megalotis*) is a freshwater fish in the sunfish family, Centrarchidae, of the order Perciformes. It is native to eastern North America from the Great Lakes down to northeastern Mexico (Fig. 15a). Six subspecies are recognized: *aquilensis*, *solis*, *ouachita*, *megalotis*, *ozark*, and *pelastes*. Due to the widespread geographic distribution and frequent hybridization, species delimitation in the longear sunfish poses considerable challenges. Kim et al. (2022) analyzed a dataset of 163 ddRAD loci (average sequence length 89 bp) sampled from 50 individuals from the 6 subspecies. After inferring a species/population phylogeny using IQ-tree, they analyzed the data under the MSC model with no gene flow using bpp to calculate gdi scores to delimit species in the group. They found that none of the population pairs had high gdi values to support distinct species status. Kim et al. (2022) also found evidence for multiple instances of historical or ongoing gene flow.

We re-analyzed the data of Kim et al. (2022), using the MSC-M model to calculate gdi, accommodating migration between the subspecies. Based on the hybridization patterns observed by Kim et al. (2022), migration from *megalotis* to *pelastes*, *solis*, and *ozark* was specified in the guide tree (Fig. 15b). Migration rates were assigned the gamma prior G(0.1, 10) with a mean of 0.01. Merge and split algorithms were run using gdi thresholds of 0.3 and 0.7 (control file in Supplementary Fig. S7). Each iteration of the algorithm took ∼20 h using 16 threads, with a total runtime of ∼120 h.

Both merge and split analyses supported a single species. This is congruent with the delimitation of Kim et al. (2022), in which gdi was calculated under the MSC model without gene flow. Estimates of the migration rates between the subspecies during the merge algorithm (Table 3) were consistently large, supporting the classification of those populations as a singlespecies.

**Table 3:** Estimates of migration rates between 5 sunfish populations during the merge algorithm (Figure 15).

It.	Donor	Recipient	M (95% HPD CI)
1	megalotis	solis	0.605 (0.412, 0.808)
	megalotis	pelastes	0.537 (0.370, 0.709)
	megalotis	ozark	0.322 (0.103, 0.596)
2	megalotis	solis	0.692 (0.462, 0.945)
	megalotis	PelOzk	0.693 (0.397, 0.989)
3	PelOzkMeg	Solis	0.579 (0.387, 0.785)
4	PelOzkMegOua	Solis	0.407 (0.279, 0.541)

## Discussion

### Heuristic Species Delimitation with Gene Flow and Paraphyletic and Polytypic Species

In this paper, we have developed a python pipeline to automate hierarchical merge and split algorithms for heuristic species delimitation. The merge algorithm was described and applied by Leaché et al. (2019), and here we have made the procedure automatic. We have also implemented the hierarchical split algorithm. Our tests using both simulated and empirical datasets suggest that the heuristic algorithms based on gdi may be less prone to over-splitting, which has been discussed extensively as a problem with the approach of Bayesian model selection implemented in bpp (Yang and Rannala 2010).

Heuristic species delimitation discussed here may be considered refinements of earlier heuristics including genetic-distance cutoffs (such as the “10× rule” in DNA barcoding, Hebert et al. 2004) and reciprocal monophyly of gene trees (Baum and Shaw 1995). For example, under the complete-isolation model (MSC with no gene flow), gdi (Equation (2)) is a simple function of τ/(θ/2)=T/(2N), which contrasts within-species polymorphism with between-species divergence, just as does the “10x rule” — note that 2N is the average divergence time (in generations) between 2 sequences sampled from within the same species (of size N) while T is the species split time (in generations). Similarly gene tree $G_{1}=\left(\left(a_{1},a_{2}\right),b\right)$ is one of within-species monophyly given the 3 sequences at the locus ($a_{1},a_{2},b$). Earlier criteria make use of simple summaries of the genetic data, whereas the methods discussed here are based on population parameters. Distinguishing data summaries from population parameters and adopting a statistical inference framework makes it easy to address properly concerns such as gene-tree reconstruction errors due to lack of phylogenetic information, stochastic fluctuations of the coalescent process across the genome, etc. Note that reliable estimation of the species tree and population parameters is possible from analysis of genomic data even if every locus contains very weak phylogenetic information (Xu and Yang 2016). Indeed simulation studies suggest that genomic data provide rich information concerning population histories, and the MSC framework is powerful to produce precise and accurate estimation of population parameters (e.g., Huang et al. 2020; Thawornwattana et al. 2022; Ji et al. 2023). As gdi is defined as a function of parameters, by definition gdi will be well estimated from genomic data as well.

Our pipeline requires the user to supply a guide tree. This may be inferred using bpp under the MSC model with no gene flow (Yang and Rannala 2014; Rannala and Yang 2017). Other programs implementing the MSC may be used as well, such as *beast (Douglas et al. 2022) and IMa (Hey et al. 2018). Phylogenetic programs such as IQ-tree (Minh et al. 2020) and RAxML (Stamatakis et al. 2012) may also be used to infer the maximum likelihood tree using concatenated genomic data or mitochondrial genomic sequences.

We note that the hierarchical merge and split algorithms implicitly assume a monophyletic species definition and thus do not work when a species is paraphyletic. Paraphyletic species, or species comprising of multiple populations that are not monophyletic, appear to be common (Crisp and Chandler 1996). Note that one may insist on higher taxa being always monophyletic while allowing for paraphyletic species (Crisp and Chandler 1996). The model tree of Figure 4a represents such a scenario, in which species ABCD is paraphyletic. The issue here concerns the non-monophyly of the populations of the same species, and is different from the monophyly of a gene tree, which is problematic if used as a criterion for species delimitation (Knowles and Carstens 2007). Non-monophyly of gene trees is a natural consequence of the coalescent process under the MSC model and can arise even if the populations of each species are monophyletic.

If all populations are completely isolated with no gene flow, the concept of a paraphyletic species does not appear to be sensible. For example, if the population phylogeny is the model of Figure 4a but without gene flow, that is, ((((X,A),B),C),D), it does not appear sensible to designate population X as a distinct species while lumping populations A, B, C, and D into 1 species, given that populations B, C, and D split from A earlier than X did. However, with gene flow between populations, the population divergence history may render the species to be paraphyletic (as in the model of Figure 4a with gene flow). In this study, we have explored 2 approaches to delimiting paraphyletic species or to accommodating gene flow during heuristic species delimitation.

The first is to use a guide tree for all populations (including those that make up the paraphyletic species) assuming no gene flow. This is used in Leaché et al. (2019, Fig. 3b) and in this paper, where the guide tree is constructed under the MSC model ignoring gene flow and then used to calculate the gdi (Fig. 4b,c). The resulting guide tree may reflect gene flow as well as population divergence (Fig. 4b,c) and may differ from the population phylogeny. For the simulated dataset of Figure 4, this led to delimitations of either 1 species at the cutoff of 0.7 or 2 species (ABCD and X) at the gdi=0.2 cutoff (see also Supplementary Table S2). The results appeared sensible even though the guide tree used did not have the correct topology.

The second approach is to use the MSC-M model accommodating gene flow in the guide tree (e.g., the MSC-M model of Fig. 4a), but allow the merge of non-sister lineages involved in gene flow in the merge algorithm (e.g., A and B; Fig. 4a). When 2 non-sister populations are merged, one may use the idea of *displayed species trees* (Degnan 2018) to generate the new species tree or model. For example, if populations B and D are merged because of high migration rate $M_{BD}$, we may merge D into B so that the species tree becomes (((X,A),(B,D)),C), whereas if $M_{DB}$ is high, we may merge B into D to give the species tree (((X,A),C),(B,D)). This approach is not yet implemented in hhsd, but we applied it manually to the simulated data of Figure 4a in Supplementary Table S3, and the results appeared sensible.

Even within the framework of Bayesian model selection, multiple approaches may be possible when there is gene flow between populations. Given populations A and B, 3 models may be considered: (i) $H_{1}$: 1 single species, (ii) $H_{2ø}$: 2 species with no gene flow, and (iii) $H_{2m}$: 2 species with gene flow (with either $M_{AB}>0$ or $M_{BA}>0$ or both). Leaché et al. (2019) compared $H_{1}$ and $H_{2ø}$ to decide whether there is 1 or 2 species, and noted that if a population split is followed by gene flow so that $H_{2m}$ is the true model, then $H_{2ø}$ is less wrong than $H_{1}$ and will win over $H_{1}$, potentially leading to over-splitting. Alternatively one may insist on species status only if there is no significant evidence for gene flow, that is, only if $H_{2ø}$ wins over both $H_{1}$ and $H_{2m}$). This may arguably be a more faithful implementation of the biological species concept (Dobzhansky 1937; Mayr 1942; Coyne and Orr 2004) than the comparison between $H_{1}$ and $H_{2ø}$ (Yang and Rannala 2010). However, this approach may lead to over-lumping since some “good” species are known to exchange migrants.

### Challenges and Utility of Heuristic Species Delimitation

The greatest challenge to heuristic species delimitation, when applied to determine the species status of allopatric geographical populations, may be the arbitrary nature of species concept (e.g., de Queiroz 2007; Mallet et al. 2023; Maddison and Whitton 2023). Even if a full characterization of the history of the populations is available, in terms of the order and timings of population splits, population sizes, and the directions, timings and strengths of gene flow between populations, a universally accepted view on species status may not exist. Darwin considered the difference between a species and a variety (subspecies, race, or population) to be one of degree, while Bateson (1909) considered species to have a “strict and concrete meaning in contradistinction to the term Variety” and suggested hybrid sterility as a test of species status. The biological species concept (Dobzhansky 1937; Mayr 1942; Coyne and Orr 2004) emphasizes reproductive isolation as the major criterion for species status. Thus, heuristic species delimitation discussed here is more in keeping with Darwin’s view that species are continuous, with fuzzy boundaries between species and “varieties” (subspecies, races, or populations). Allopatric populations that do not overlap in their geographical distributions, with no or little gene flow between them, may be classified as distinct species, or subspecies of a polytypic species, and some arbitrariness appears unavoidable.

The large interval of uncertainty for gdi: 0.2<gdi<0.7 (Jackson et al. 2017) should be considered a consequence of the arbitrariness of the heuristic delimitation. This is also the main cause for different species delimitations in analysis of the same data using the same guide tree by the merge and split algorithms, as in our analyses of the giraffe and milksnake datasets (Figs. 13c,d and 14c,d). The cutoffs of Jackson et al. (2017) were based on estimates of population parameters in 178 empirical studies compiled by Pinho and Hey (2010, Supplementary Table S1). The datasets analyzed in those studies were small, mostly with only a few loci for 2 populations, and the summaries were medians of estimates in major taxonomic groups. It may be profitable to redo the meta-analysis, using more recent genomic sequence data and improved analytical methods to generate empirical estimates of population parameters in well-studied systems where the species status of the populations is well established. Such an effort may be hoped to lead to refined criteria and cutoffs (with reduced interval of uncertainty).

In our hierarchical algorithms, it should be straightforward to use empirical criteria other than the gdi. It is also possible to apply a composite criterion; for instance, besides the gdi cutoff, we may require a minimum species split time (in generations or in years) (Rannala and Yang 2020). When there exist contact zones between populations, one may estimate the proportion of hybrids (h) (Anderson and Thompson 2002; Chakraborty and Rannala 2023), and contrast it with the historical migration rate (m) estimated from genomic data (Beerli 2006; Hey 2010; Hey et al. 2018; Gronau et al. 2011; Flouri et al. 2023). The rate ratio m/h may be used to measure reproductive isolation: a value of 1 means that introgressed alleles are neutral and have the same chance of being retained as a native allele in the recipient population, while m/h≪1 means that introgressed alleles are strongly deleterious and purged from the population by natural selection, indicating the existence of (post-zygotic) reproductive isolation (Westram et al. 2022). A composite criterion incorporating m/h may be informative about species status, although a strict adherence to reproductive isolation (i.e., m/h=0) as the criterion for delimiting species may be untenable given the prevalent nature of gene flow between well-recognized species.

While acknowledging the caveats of empirical species delimitation, we suggest that our pipeline allows one to utilize the power of the MSC framework and the bpp program to estimate population parameters precisely and accurately using the ever-increasing genomic sequence data. In particular, the recent implementation of the MSC-M model in bpp, having been applied to genome-scale datasets with thousands of loci (Flouri et al. 2023; Thawornwattana et al. 2022, 2023), has greatly improved the biological realism of models that are available for analyzing genomic data from closely related species and populations, the species status of which is yet to be determined. We hope that our pipeline may become a useful tool for evolutionary biologists to assess the genetic evidence for species delimitation, which should be integrated with other lines of evidence, including morphological and behaviorial characteristics, and patterns of hybridization (Fujita et al. 2012; Solis-Lemus et al. 2015; Kim et al. 2022).

## Data Availability

The hhsd pipeline is written in python, and drives parameter estimation under the MSC or MSC-M models using bpp. The source code, documentation, and empirical datasets analyzed in the paper are available at https://github.com/abacus-gene/hhsd.

## Supplementary Material

Data available from the Dryad Digital Repository: https://doi.org/10.5061/dryad.jm63xsjhc.
